# Spatio-temporal genetic tagging of a cosmopolitan planktivorous shark provides insight to gene flow, temporal variation and site-specific re-encounters

**DOI:** 10.1038/s41598-020-58086-4

**Published:** 2020-02-03

**Authors:** Lilian Lieber, Graham Hall, Jackie Hall, Simon Berrow, Emmett Johnston, Chrysoula Gubili, Jane Sarginson, Malcolm Francis, Clinton Duffy, Sabine P. Wintner, Philip D. Doherty, Brendan J. Godley, Lucy A. Hawkes, Matthew J. Witt, Suzanne M. Henderson, Eleonora de Sabata, Mahmood S. Shivji, Deborah A. Dawson, David W. Sims, Catherine S. Jones, Leslie R. Noble

**Affiliations:** 1 School of Biological Sciences, University of Aberdeen, Zoology Building, Tillydrone Avenue, Aberdeen, AB24 2TZ Scotland UK; 2 Manx Basking Shark Watch and Manx Wildlife Trust, Peel, Isle of Man, IM9 5PJ UK; 3 Irish Basking Shark Study Group, Merchants Quay, Kilrush, County Clare UK; 4 Marine and Freshwater Research Centre, Galway-Mayo Institute of Technology, Dublin Road, Galway, Ireland; 5 School of Biological Sciences, Queen´s University Belfast, Belfast, Northern Ireland UK; 6 Hellenic Agricultural Organisation, Fisheries Research Institute, Nea Peramos, Kavala, Macedonia 64007 Greece; 7 National Institute of Water and Atmospheric Research, Private Bag 14901, Kilbirnie, Wellington, 6241 New Zealand; 8 Department of Conservation, Private Bag 68908, Wellesley Street, Auckland, 1141 New Zealand; 9 KwaZulu-Natal Sharks Board, Private Bag 2, Umhlanga Rocks, 4320 South Africa; 10 School of Life Sciences, University of KwaZulu-Natal, Durban, 4000 South Africa; 11 Centre for Ecology and Conservation, University of Exeter, Cornwall Campus, Penryn, TR10 9FE UK; 12 Environment and Sustainability Institute, University of Exeter, Cornwall Campus, Penryn, TR10 9FE UK; 13 Scottish Natural Heritage Great Glen House, Inverness, IV3 8NW Scotland UK; 14 MedSharks, via Ruggero Fauro 82, 00197 Rome, Italy; 15 Save Our Seas Shark Research Center and Guy Harvey Research Institute, Nova Southeastern University, 8000 North Ocean Drive, Dania Beach, FL 33004 USA; 16 NERC Biomolecular Analysis Facility, Department of Animal and Plant Sciences, University of Sheffield, Sheffield, S10 2TN South Yorkshire UK; 17 Marine Biological Association of the United Kingdom, The Laboratory, Citadel Hill, Plymouth, PL1 2PB UK; 18 Ocean and Earth Science, National Oceanography Centre Southampton, University of Southampton, Waterfront Campus, Southampton, SO14 3ZH UK; 19 Faculty of Science and Engineering, John Dalton Building, Manchester Metropolitan University, Chester Street, Manchester, M1 5GD UK; 20 School of Chemistry and Chemical Engineering, Queen´s University Belfast, Marine Laboratory, Portaferry, BT22 1PF Northern Ireland UK; 21 Faculty of Biosciences and Aquaculture, Nord University, Postboks 1490, 8049 Bodø, Norway

**Keywords:** Animal behaviour, Molecular ecology

## Abstract

Migratory movements in response to seasonal resources often influence population structure and dynamics. Yet in mobile marine predators, population genetic consequences of such repetitious behaviour remain inaccessible without comprehensive sampling strategies. Temporal genetic sampling of seasonally recurring aggregations of planktivorous basking sharks, *Cetorhinus maximus*, in the Northeast Atlantic (NEA) affords an opportunity to resolve individual re-encounters at key sites with population connectivity and patterns of relatedness. Genetic tagging (19 microsatellites) revealed 18% of re-sampled individuals in the NEA demonstrated inter/multi-annual site-specific re-encounters. High genetic connectivity and migration between aggregation sites indicate the Irish Sea as an important movement corridor, with a contemporary effective population estimate (*N*_*e*_) of 382 (CI = 241–830). We contrast the prevailing view of high gene flow across oceanic regions with evidence of population structure within the NEA, with early-season sharks off southwest Ireland possibly representing genetically distinct migrants. Finally, we found basking sharks surfacing together in the NEA are on average more related than expected by chance, suggesting a genetic consequence of, or a potential mechanism maintaining, site-specific re-encounters. Long-term temporal genetic monitoring is paramount in determining future viability of cosmopolitan marine species, identifying genetic units for conservation management, and for understanding aggregation structure and dynamics.

## Introduction

The movement and spatio-temporal patterns of large, mobile marine predators can be highly dynamic with frequent, ocean-scale movements prevalent among cetaceans, seabirds, turtles, teleost fish, and sharks^1^. Gaining insight into repeated long-distance movement, its drivers and evolutionary consequences, is fundamental to understanding the ecology of these species. It is also central in evaluating current environmental concerns during an era of rapid climate change and extensive marine habitat modification, to inform the management and design of effective networks of marine protected areas (MPAs).

Chondrichthyan (sharks, skates, chimaeras) extinction risk is substantially higher than for most other vertebrates, particularly for large–bodied sharks frequenting coastal waters^2^. The filter-feeding basking shark, *Cetorhinus maximus*, and the whale shark, *Rhincodon typus*, are the world’s largest fish. Their horizontal movement is primarily driven by zooplankton dynamics and typically exceeds thousands of kilometres, precluding knowledge of natal origins and the potential existence of mating and breeding areas for either of these planktivores^3,4^. Identifying recurring spatial patterns, such as seasonal migration between distinct habitats or behavioural fidelity to ecologically important sites (feeding, breeding or mating areas) therefore remains a challenge.

While site fidelity, returning to an area over time, has been noted in more than 30 elasmobranch species^5^, only a few telemetry studies have achieved multi-year tracks to detect migratory behaviour^6^. Migration, typically defined as the more persistent, seasonal movement of animals from one place to another, is often driven by transitory availability of resources^7^ and may play an especially important role in shaping the spatial ecology of planktivores given their dependence on such highly heterogeneous prey distributions.

The basking shark is circumglobally distributed in temperate seas, but the western European shelf provides key habitats supporting relatively predictable, seasonal coastal aggregations between April and September off Southwest England, Ireland, Northwest Scotland and the Isle of Man (Irish Sea)^8–11^. There is some evidence of courtship within aggregations^11^, but general timing and areas for breeding or pupping have yet to be determined, with the possibility that these life history events are less structured in time and space compared with other sharks^12^. Basking sharks forage selectively on zooplankton^13^, and within the Northeast Atlantic (NEA) generally use deeper waters near shelf edges during winter, forming surface/temporal aggregations in shallower coastal waters during summer^14^.

In addition to extensive basking shark movement across the European shelf, there is evidence for ocean-scale transits, both trans-Atlantic^15^ and trans-equatorial^16^. Increasingly, satellite telemetry suggests that these latitudinal, long-range movements are less likely to be random (i.e. dispersal), but instead represent seasonal movement patterns, characteristic of migration^14,17^. In the NEA, most documented return migrations are to the general vicinity of the Exclusive Economic Zone (EEZ) of UK and Irish waters^17^, with two tagged sharks returning to within 30 km of the previous centre of activity, within the area encompassing a proposed Scottish MPA^18^. As an adaptation to the seasonal, temperate habitat of the NEA, basking sharks may exhibit seasonal migration patterns where inter-annual site-fidelity to aggregation sites with elevated zooplankton densities^17,19^ (‘hotspots’ hereafter) could have genetic consequences, such as spatial patterning in genetic connectivity. Investigating the existence, extent and persistence of such patterns is fundamental to understanding the consequences of migratory behaviour.

Contrasting with the temporary attachment of satellite tags, the permanence of genetic identity enables lifelong “tracking” of individuals, resampling of which can indicate site fidelity^20^. Through repeated genetic sampling of basking sharks at key sites, questions pertaining to multiannual site fidelity and genetic connectivity can be addressed in the context of species management measures, such as assessing the spatial adequacy of MPAs.

While temporary aggregations may constitute an appreciable proportion of a NEA population, it has not been established if they represent genetically distinct management units, or a random genetic assemblage of a wider, global population, spatially drawn together by prey availability. Nonetheless, the prevailing perspective is that basking sharks lack global population structure and display low worldwide genetic variability^21^. However, this earlier study was limited by a small number of opportunistic samples and the use of a single, maternally-inherited genetic marker (mtDNA). In contrast, the use of bi-parentally inherited markers, such as microsatellites, allows a more contemporary genetic assessment of connectivity and population structure. Although evaluating demographic independence among marine populations using genetic markers remains challenging^22^, it can assist in the identification of conservation management units^23^. With analyses of genetic structure influenced by marker properties, models of mutation, selection and sample size, finding a lack of structure can seldom be regarded as definitive^24^. For instance, in the whale shark, the assumption of global panmixia using mtDNA was revised when microsatellite markers indicated genetic differentiation between Indo-Pacific and Atlantic populations^25^.

Further, genetic monitoring can identify temporal changes in population genetic metrics affecting population viability^26^, including effective population size (*N*_*e*_), the number of breeding individuals within a population; an evolutionary analogue of adult census size^27^. This parameter complements abundance estimates and is critical for informed long-term conservation management. Formerly extensively harvested as a commercial species, basking sharks are classified as ‘Endangered globally’^28^ (IUCN 2018). In spite of these concerns, no long-term regional population abundance estimates are available in the NEA due to the low number of re-sightings. In an attempt to estimate local abundance through mark-recapture in the NEA, a closed population model (assuming demographic closure) was applied previously, generating an estimate of 985 sharks in a 50 km diameter study area^29^.

To date, the population genetic consequences (e.g. gene flow, diversity) of the basking shark’s complex migration strategies^17^ remain only poorly understood. Here, we use a panel of recently developed, species-specific microsatellite loci^30^ to test the null hypothesis of global panmixia using >400 basking shark samples collected worldwide. Secondly, using temporal samples collected at three known NEA aggregation sites from 2009 to 2014 inclusive, we assess genetic re-encounters, contemporary gene flow and migration rates between sites to infer regional connectivity, complementing and expanding previous studies using mtDNA^21^. Further, we provide a preliminary examination of average within-group relatedness to determine if aggregations are on average more related than expected by chance. Finally, we provide the first estimates of effective population size to assess contemporary *N*_*e*_ at a time when increasing anthropogenic activity (e.g. marine renewable energy installations, fishing, shipping, and oil and gas extraction) is rapidly changing the NEA seascape.

## Results

### Microsatellite characterisation

A minimum of 13 out of 22 microsatellite loci were successfully amplified in the 460 DNA samples screened, which included 394 samples from the NEA (divided into IRE = Ireland, IoM = Isle of Man, MF = Moray Firth, SCO = West Scotland, S_ENG = South England) collected over 6 years (2009–2014) and 66 samples opportunistically collected over 20 years from elsewhere in the world (MED = Mediterranean, NWA = Northwest Atlantic, PAC = New Zealand, SA = South Africa). Genotypes of individuals sexed at the time of sampling (78 females and 69 males) suggested all 22 microsatellites behaved as autosomal, co-dominant loci. Duplicate individuals (n = 53 comprising of NEA n = 51, SA n = 1, NWA n = 1 following matching analysis) were removed, leaving a total of 407 unique individuals to characterise the microsatellites. Initial and final sample sizes can be found as Supplementary Table S1.

None of the loci were out of Hardy Weinberg equilibrium (HWE) overall (across populations). When HWE was assessed within global putative populations based on geographic origins (for putative populations and sample sizes see Supplementary Table S2), significant departures were identified for locus *Cmax07* in three out of nine populations. None of the loci showed any evidence for stuttering, large allelic dropout or the presence of null alleles, except for *Cmax12*. However, *Cmax13 and Cmax14* displayed significant linkage disequilibrium with several loci (*Cmax06, Cmax07, Cmax17*). Therefore, *Cmax12*–*Cmax 14* were removed and all following analyses performed with the 19 validated loci.

The genotyping error rate was 0.0097 (i.e. 16 incorrect genotypes/1635 reprocessed genotypes), where the error rate per locus ranged from 0–0.0230 (see Supplementary Table S3). When confirming re-encounters, genetic match #3 (Table 1) included two mismatching loci resulting most likely from large allelic dropout in *Cmax04* and a null allele causing a false homozygote in *Cmax05*, whereas genotyping errors in match #5 were probably due to large allele dropout at loci *1HC2* and *1HF4*, where the larger allele failed to amplify in the more degraded sample. In both cases, the more degraded sample (which showed homozygous alleles) was removed following matching analyses. Across all 407 individuals, the number of alleles per microsatellite locus ranged from 3 to 18 with a mean number of alleles per locus of 8 (mean proportion of individuals typed = 0.98). Observed and expected heterozygosity per microsatellite ranged from 0.22 to 0.87 (mean = 0.66) and 0.24 to 0.87 (mean = 0.66), respectively (see Supplementary Table S3).

**Table 1:** Inter-annual genetically confirmed re-encounters.

Re-encounter	Site	Sample ID	Sex	Date sampled	# mismatching loci	Interval (days)	Distance (km)
1	IoM	IoM09_01	Male	07.08.2009	0	323	4.5
	IoM	IoM10_03	Male	26.06.2010			
2	IoM	IoM09_06	Female	09.08.2009	0	1395	18.2
	IoM	IoM13_02	Unknown	04.06.2013			
3	IoM	IoM11_09	Male	03.08.2011	2	358	2.4
	IoM	IoM12_01	Male	26.07.2012			
4	IoM	IoM10_08	Female	26.06.2010	0	775	12.8
	IoM	IoM12_24	Female	09.08.2012			
5	IoM	IoM10_18	Female	23.07.2010	2	748	5.4
	IoM	IoM12_19	Unknown	09.08.2012			
6	IRE_12	IRE11_21	Unknown	29.04.2011	0	339	1.6
	IRE_12	IRE12_04	Unknown	02.04.2012			
7	SCO	SCO12_08	Unknown	08.08.2012	0	344	3.8
	SCO	SCO13_22	Unknown	18.07.2013			

**Figure 1:**
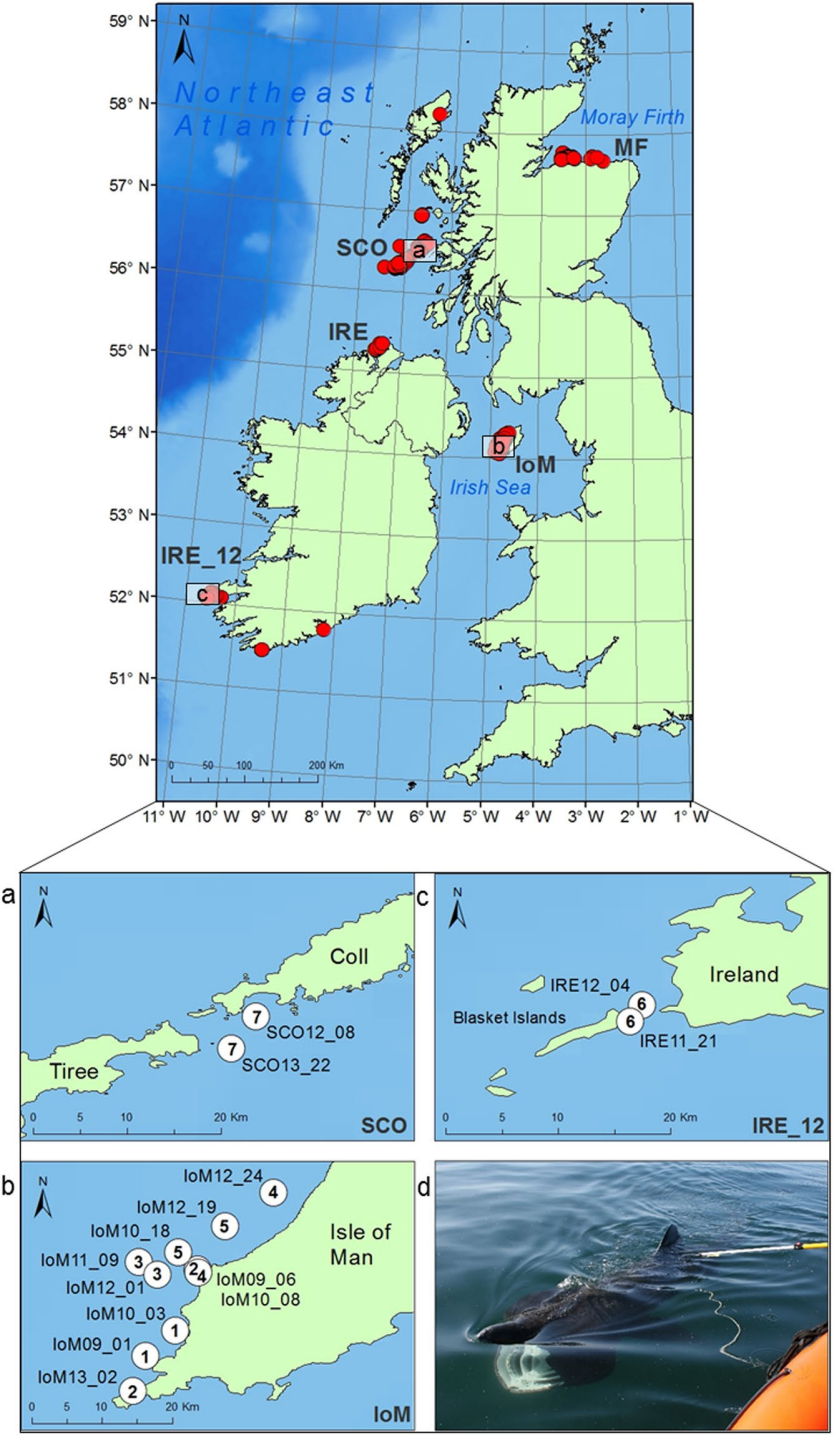
Basking shark NEA sampling sites and inter-annual, genetically confirmed re-encounters. Approximate basking shark sampling sites (red circles) from the Northeast Atlantic collected 2009–2014, including the Moray Firth (MF) and common aggregation sites around Ireland (IRE, IRE_12), Scotland (SCO) and the Isle of Man (IoM). (**a**–**c**) Locations of the seven inter-annual, genetically-confirmed re-encounters (see Table 1). The map was created in ArcGIS v.10 (http://www.esri.com/arcgis/about-arcgis), bathymetry reproduced from GEODAS (Geophysical Data system) Grid Translator developed by the National Geophysical Data Center, NOAA (National Oceanic & Atmospheric Administration). (**d**) Image of basking shark mucus sampling off the North Coast of Ireland. Photo credit: S. Berrow.

Individuals resampled at the same site showing pairings that matched at all or nearly all 19 microsatellite loci, demonstrating exact inter-annual site-fidelity (<20 km) in basking sharks sampled around the Isle of Man (IoM), Ireland (IRE_12) and the West Coast of Scotland (SCO). Re-encounter numbers refer to mapped shark locations displayed in Fig. 1(a–c). Distance refers to the euclidean distance measured between sampling sites, while interval refers to the days in between sampling occasions.

Two loci possibly under positive selection (*Cmax16* and *Cmax17*) and three loci (*Cmax01, Cmax06 & 1HF4*) under balancing selection were identified. However, the significance of pairwise estimates of population differentiation remained unchanged whether non-neutral loci were either included or excluded, justifying their incorporation in all subsequent analyses to maximize resolution.

Based on 407 individuals, the probability of two individuals sharing the same multi-locus genotype across all 19 loci was very low with P_ID_(unbiased) = 5.25 × 10^−17^ and P_ID_(sibs) = 3.11 × 10^−07^, indicating the high discriminatory power of this marker set.

### Inter-annual returns to northeast Atlantic aggregation sites

There were 51 ‘genetically confirmed re-encounters’ (individuals sampled repeatedly) in the NEA region throughout the course of this study. Of these, 82% (n = 42) occurred within the same site and year (seasonal residency), mostly within a day or week (max. 31 days) of the first sampling (see Supplementary Fig. S1).

The remaining 18% (n = 9) were re-sampled in different years at the same or different localities within the NEA. Two of these individuals were re-sampled at locations >100 km distant from their original site and represent inter-annual site fidelity on a regional scale. One individual, first sampled in 2012 on the Scottish West Coast, was re-sampled off Ireland (north) two years later (SCO12_60 & IRE14_06). Another, originally encountered off the Scottish West Coast (SCO12_42) in 2012 (7 m, male), was re-sampled in the Moray Firth in 2013 (MF13_11).

The remaining 7 individuals (14%) resampled at the same locality (i.e. within 20 km of the original site) between years, are indicative of inter-annual site-fidelity (Table 1; Fig. 1). They comprise five IoM individuals (out of 118 samples collected 2009–2013), another from SCO (from Gunna Sound) (out of 123 samples collected 2012–2014), and one from IRE (out of 124 samples collected 2009–2012, and in 2014). The latter was sampled off Ireland during April of two successive years (2011 & 2012). Of those re-encounters where individuals could be sexed, those identified as males were sampled in successive years (n = 2), whereas those confirmed as females (n = 3) were sampled after an interval of two to four years.

### Northeast Atlantic population differentiation and gene flow

None of the pairwise comparisons of *D*_ST_ (105 possible pairwise comparisons of 15 temporal populations = 5 putative populations at aggregation sites split by year) remained significant (95% confidence intervals bounded by zero) following bootstrapping. All but three *G*_ST_ pairwise values were initially significant, yet only the following comparisons involving IRE_12 (early season samples at the geographic periphery of our NEA sampling range, collected off SW Ireland in 2012) remained significant following bootstrapping, IoM_10 (*G*_ST_ = 0.014, CI = [0.003; 0.030]), IoM_12 (*G*_ST_ = 0.010, CI = [2.5 × 10^−5^; 0.023]) and SCO_13 (*G*_ST_ = 0.009, CI = [7.6 × 10^−5^; 0.020]) (Supplementary Fig. S2). No pairwise *F*_ST_ comparisons remained significant following Bonferroni adjustment of significance levels (Supplementary Table S4). However, IRE_12 retained the highest degree of genetic differentiation (*F*_ST_ ranging from 0.0112–0.032).

**Figure 2:**
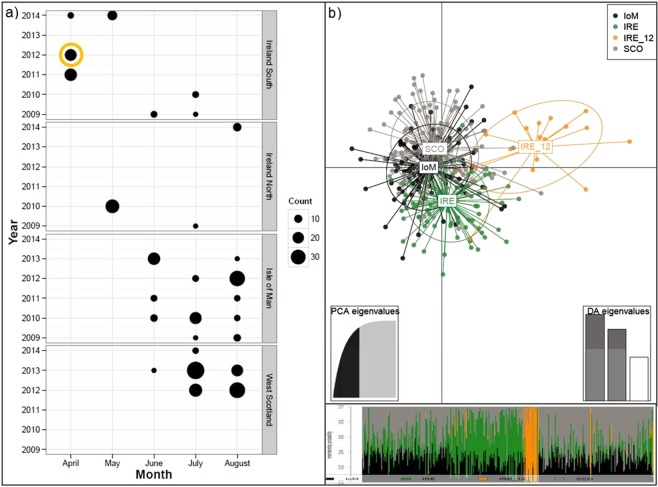
Northeast Atlantic (NEA) basking shark sampling occasions and DAPC results. (**a)** NEA sampling sites and numbers per year and month (2009–2014) with an orange circle emphasizing samples collected relatively early in the season, i.e. IRE_12. (**b)** Scatterplot from the discriminant analysis of principal components (DAPC) on NEA samples. Groups are shown as different colours and inertia ellipses, while dots represent individuals. Groups include temporally merged individuals from SCO = Scotland, IoM = Isle of Man, IRE = Ireland, and IRE_12 = Irish samples from 2012, respectively. The bottom inset is the DAPC’s ‘compoplot’, a bar plot showing the probabilities of assignment of individuals to the different, pre-defined groups (individuals are plotted along the x-axis and membership probabilities along the y-axis). Colours in ‘compoplot’ correspond to colours in the DAPC scatterplot.

Overall, Irish samples were differentiated from other NEA sites, shown in the Principal Coordinates Analysis (PCoA; Supplementary Fig. S3), with IRE_12 again the most distinct. The majority of IRE samples were collected relatively early in the season, in April and May, whereas IoM and SCO samples were obtained in late summer, from June until August (Fig. 2a). Finally, a Discriminant Analysis of Principal Components (DAPC) plot of NEA samples indicated IoM and SCO sharks were genetically similar, whereas IRE sharks, specifically IRE_12, remained genetically more distinct (Fig. 2b).

### High regional movements in the northeast Atlantic

**Figure 3:**
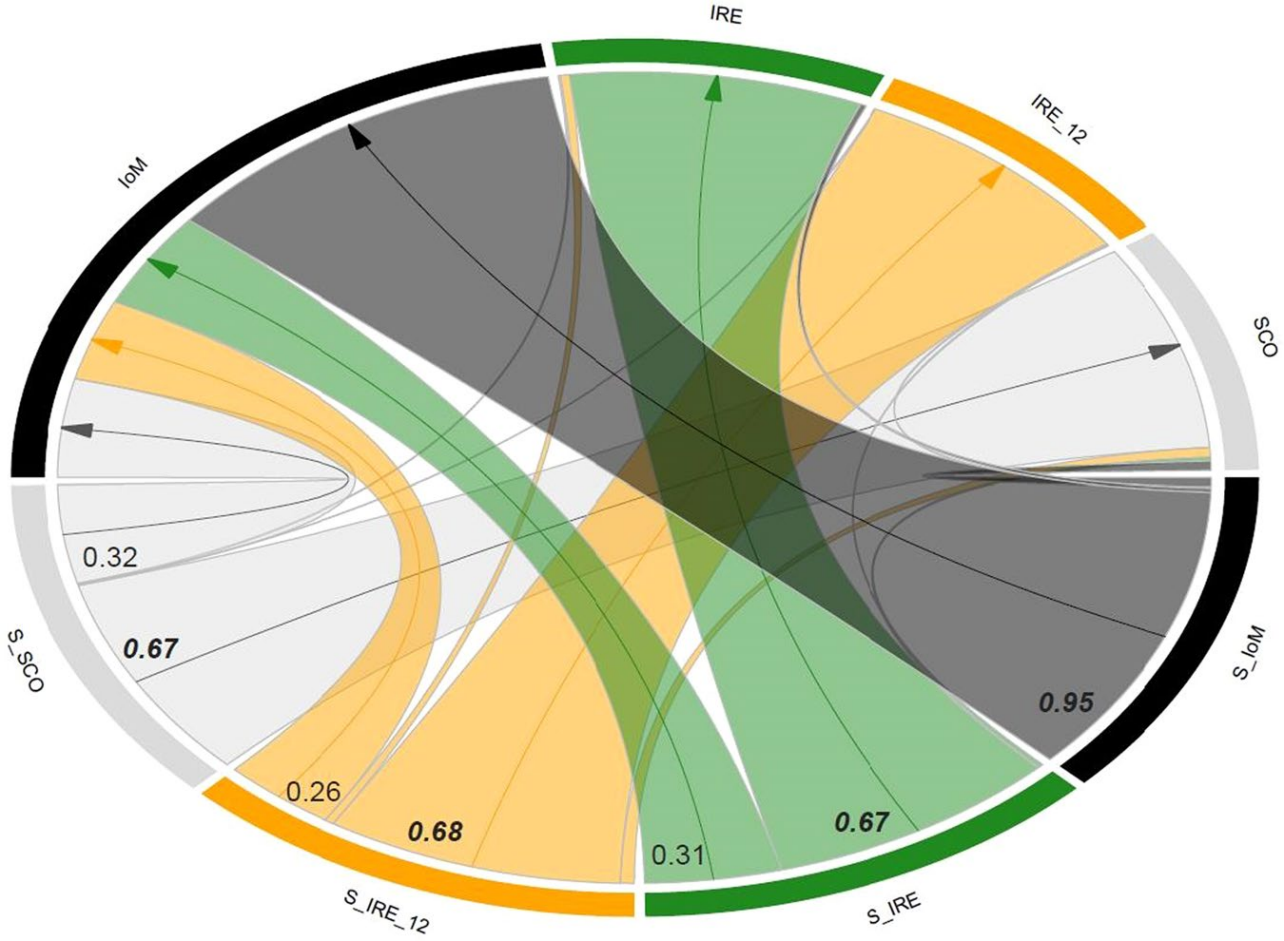
Circular migration plot (chord diagram). Migration rates (**m**) are plotted between the Isle of Man (IoM), Ireland (IRE), Scotland (SCO) and the temporal samples from Ireland 2012 (IRE_12), based on the output matrix from BayesAss. Sampling locations are given on the outside of the circle, where “S” preceding the location denotes the source population from which individuals migrated. The thickness of the arcs represents the rate of migration; only migration rates values > 0.10 are listed and indicated with an arrow. Values in bold and italic are the proportions of individuals derived from the source populations.

Analyses of contemporary migration rates (m) suggested relatively high and uni-directional movement into the Irish Sea (see IoM in Fig. 3), with mean migration rates from the three remaining sub-populations ranging from 0.26–0.32 (Supplementary Table S5, also for all mean posterior probabilities of m). The posterior probability of the population inbreeding coefficient, F, was near zero for the Irish Sea (IoM, F = 0.0174), yet relatively high for the remaining sub-populations (0.1614–0.3589). In accordance with discriminant analysis (e.g. Fig. 2b), mean migration rates from the source populations to the Irish Sea (IoM) were highest from SCO, followed by IRE and then IRE_12.

### Relatedness in northeast Atlantic aggregations

**Figure 4:**
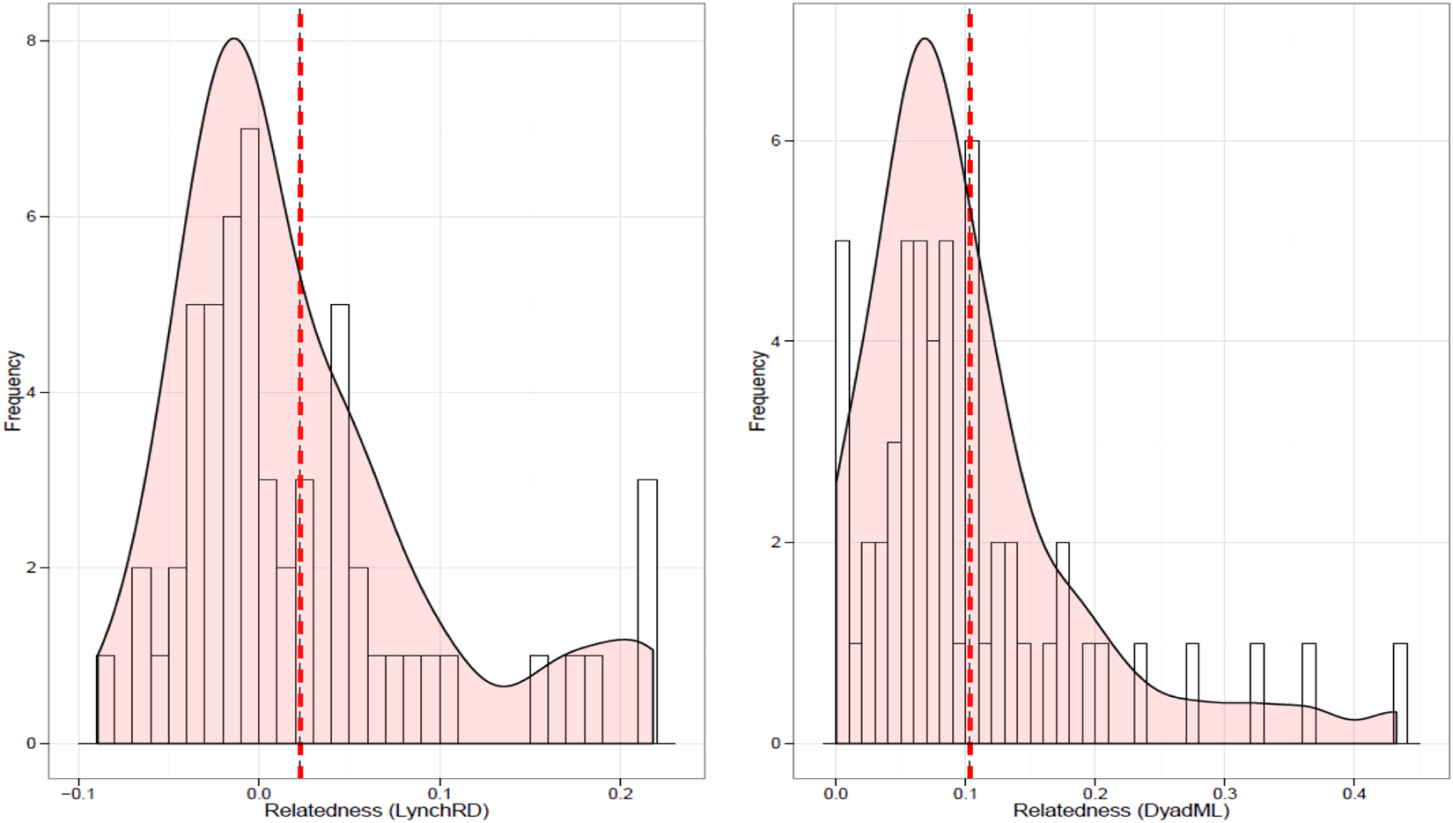
Histogram with kernel density curve of observed within-group relatedness of basking shark groups (n = 55) based on LynchRD (left; with the mean 0.0228 shown as a red dashed line) and the likelihood method DyadM (right; with the mean 0.1032 shown as a red dashed line). Groups were sampled around the Isle of Man (IoM, 2009–2013), Ireland (IRE, 2010–2012, 2014), the Moray Firth, Scotland (MF, 2013) and the West Coast of Scotland (SCO, 2012–2014).

**Figure 5:**
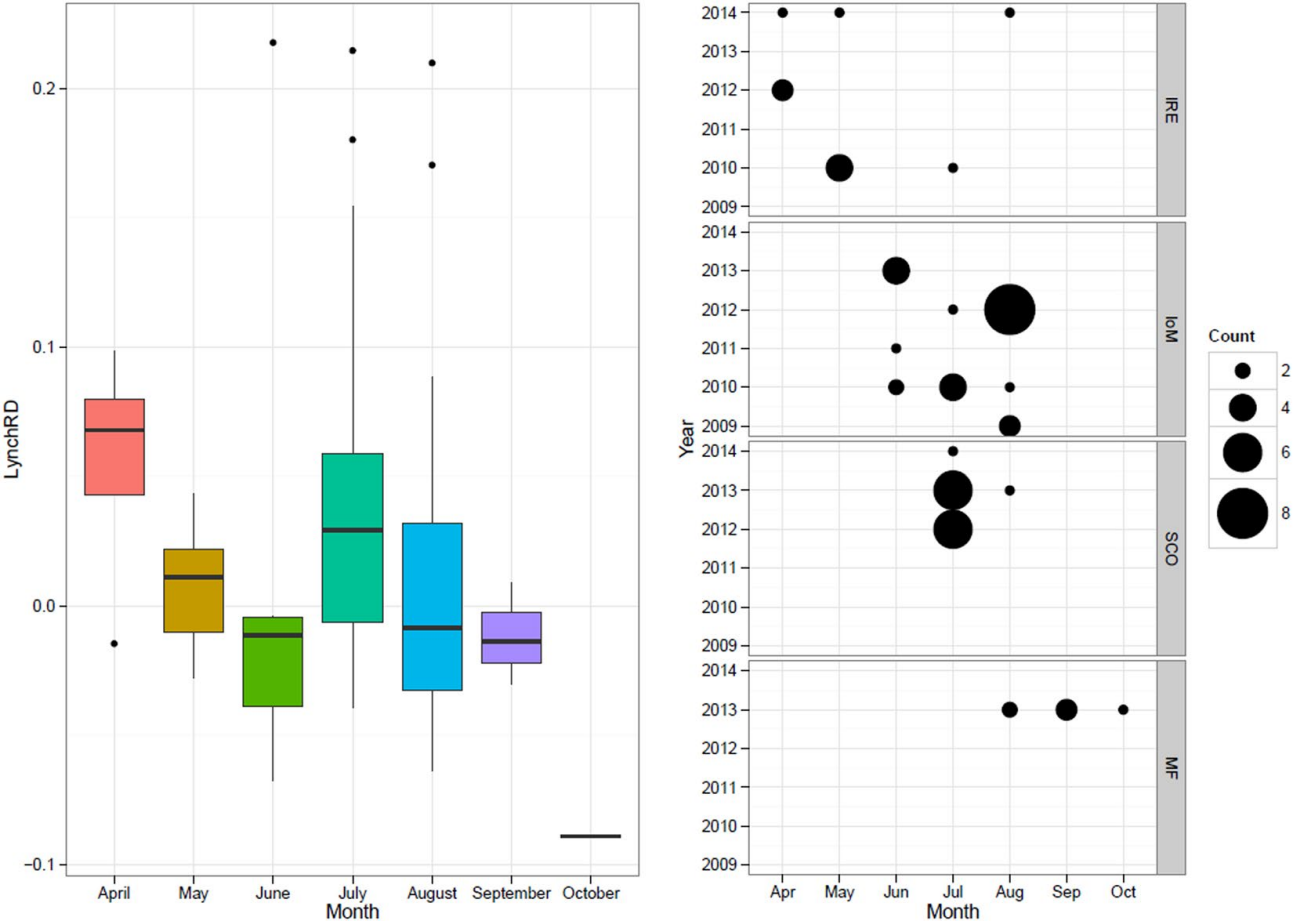
Boxplots showing the variance in within-group relatedness of basking shark groups (n = 55) per month (**a**), with the number of groups shown by site as ‘Count’ (**b**) based on LynchRD. Median relatedness was higher in samples from Ireland (**a**) and in April and July. (**b**) Sampling sites include the Isle of Man (IoM, 2009–2013), Ireland (IRE, 2010–2012, 2014), the Moray Firth, Scotland (MF, 2013) and the West Coast of Scotland (SCO, 2012–2014).

Relatedness amongst individuals of both IRE_12 (r = 0.096) and SCO (r = 0.009) was higher than other ‘population’ samples (Table 2), prompting a more detailed relatedness analysis of 55 documented adult groups within IoM, SCO, and IRE samples (Supplementary Fig. S5). Among-group relatedness was compared and assessed relative to random expectations, computed through iterations based on the re-shuffling of individuals between groups. Both the LynchRD moment-estimator and DyadML dyadic likelihood method indicated individuals within groups were more related than expected by chance. Within group relatedness was relatively high (mean of 0.0228 for LynchRD and 0.1032 for DyadML), with the right-skewed distributions of estimators indicating positive relatedness values exceeding the null distribution (Fig. 4). Within-group relatedness and within-group inbreeding for either estimator were not correlated. Monte Carlo simulations suggest the observed average relatedness calculated from LynchRD (R = 0.0228) was significantly (p < 0.005) higher than expected (R = −0.0046), indicating non-random relatedness in basking shark groups. This was supported by DyadML, both with (average observed R = 0.0775 vs expected R = 0.0521, p < 0.008, Supplementary Fig. S6) and without allowing for inbreeding (average observed R = 0.1032 vs expected R = 0.0797, p < 0.039). Comparing range of relatedness values by sampling origin showed groups of sharks sampled off Ireland had a higher median relatedness than those sampled elsewhere (Fig. 5a). Although confounded by site and within-group composition, relatedness was highest in April, with animals sampled from Ireland (IRE_12 & IRE_14) (Fig. 5b). Relatedness decreased in subsequent months, but another smaller July peak comprised sharks mainly from Scotland and the Isle of Man. In addition to the higher than expected average relatedness, both approaches identified the same five groups, and two different additional ones, suggesting each analysis supports up to seven of the 55 groups (>12%) as significantly more related than expected (Supplementary Fig. S5). With the caveat of small sample size, all-female groups (n = 5) showed higher relatedness than all-male (n = 5) or mixed gender groups (n = 45), suggesting gender-specific differences (Supplementary Fig. S7).

**Table 2:** Genetic diversity statistics (with means ± standard errors) derived from 19 microsatellite loci across nine global samples based on 407 unique individuals.

Sampling site	N	N_dA_	N_A_	P_A_	A_R_	N_E_	H_O_	H_E_	HWE	F_IS_	r
IoM	97	126	6.632	0.053	4.165	3.587	0.677	0.659	0.623	−0.029	−0.003
IRE	89	133	7.000	0.316	4.211	3.593	0.660	0.664	**0.047**	0.007	−0.011
IRE_12	18	98	5.158	0.053	4.042	3.117	0.658	0.609	0.106	−0.064	**0.096**
SCO	133	137	7.211	0.263	4.212	3.626	0.645	0.652	0.172	0.012	**0.009**
S_ENG	6	69	3.632	0.000	NA	2.844	0.693	0.593	0.998	−0.170	0.029
MED	11	90	4.737	0.053	4.295	3.300	0.639	0.645	0.717	0.027	−0.033
SA	4	64	3.368	0.000	NA	2.799	0.719	0.578	1.000	−0.226	−0.001
PAC	38	124	6.526	0.105	4.260	3.473	0.622	0.649	**0.026**	0.051	0.005
NWA	11	78	4.105	0.053	NA	3.040	0.722	0.612	0.509	−0.175	0.032
Total mean	**45**	**102.1**	**5.374**	**0.099**	**4.197**	**3.264**	**0.671**	**0.629**		−**0.063**	**0.014**
SE	±**16**	±**10**	±**0.501**	±**0.038**	±**0.700**	±**0.109**	±**0.012**	±**0.010**		±**0.034**	±**0.012**

Global sampling locations include: IoM = Isle of Man, IRE = Ireland, IRE_12 = Ireland (from 2012 only) SCO = West Scotland and the Moray Firth, S_ENG = South England, MED = Mediterranean; SA = South Africa; PAC = New Zealand, NWA = Northwest Atlantic; N, sample size; N_dA_, number of different alleles, N_A_, mean number of alleles across 19 loci; P_A_, number of private alleles; A_R_, allelic richness based on 7 diploid individuals; N_E_, number of effective alleles; H_O_, observed heterozygosity; H_E_, expected heterozygosity; HWE, p-value for Hardy-Weinberg equilibrium probability test; F_IS_, Fixation index (average inbreeding coefficient of subpopulations relative to the total population), r = mean group relatedness where bold indicates if different across populations determined by 999 permutations. NA = Non-applicable (samples excluded from allelic richness estimate due to low sample size or missing genotypes).

### High gene flow and limited global genetic differentiation

Globally, estimates of *G*_ST_ and *D*_ST_ identified weak genetic structuring among the nine putative populations (Supplementary Fig. S8), where values ranged from −0.012–0.013 and from −0.024–0.011, respectively. Only *G*_ST_ values remained significant after bootstrapping, including IRE_12 and IoM (*G*_ST_ = 0.010, CI = [0.002; 0.023]), IRE_12 and IRE (*G*_ST_ = 0.008, CI = [0.001; 0.019]), IRE_12 and SCO (*G*_ST_ = 0.009, CI = [0.001;0.020]) and IRE and SCO (*G*_ST_ = 0.002, CI = [1.2 × 10^−5^; 0.004]; for *F*_ST_ comparisons see Supplementary Table S7).

**Figure 6:**
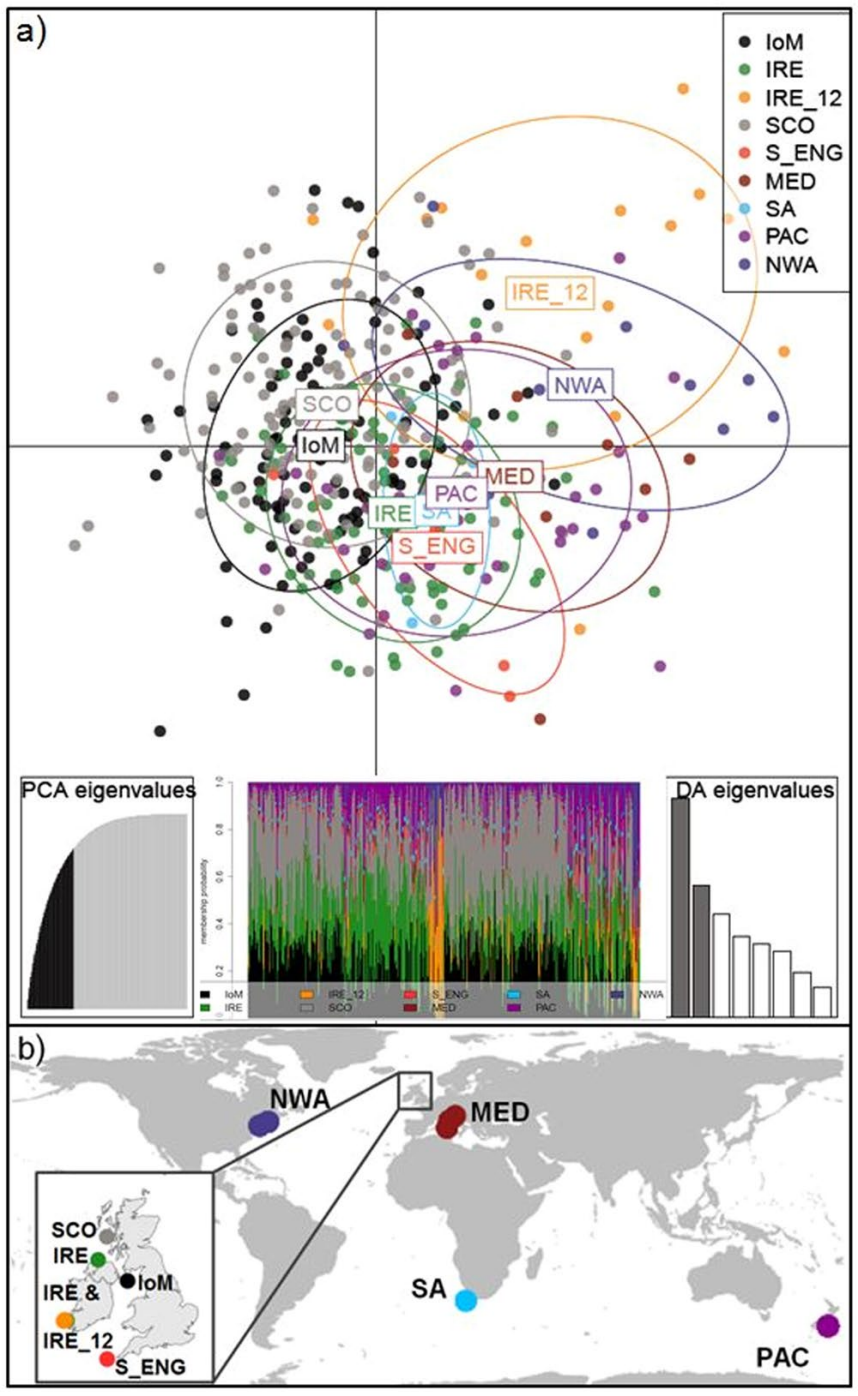
Global DAPC results and sampling locations. (**a**) Scatterplot from the discriminant analysis of principal components (DAPC) on global samples. Groups are shown as different colours and inertia ellipses (67% of variance), while dots represent individuals. The left inset indicates the number of retained principal components (60), cumulatively explaining 80% of the variance. The middle inset is the DAPC’s ‘compoplot’, a bar plot showing the probabilities of assignment of individuals to the different, pre-defined groups (individuals are plotted along the x-axis and membership probabilities along the y-axis). Colours in ‘compoplot’ correspond to colours in the DAPC scatterplot. The right bar graph inset indicates the amount of variance explained by the two discriminant eigenvalues used for plotting. (**b**) Map of global sampling locations with the Northeast Atlantic samples as an inset; where IoM = Isle of Man; IRE = Ireland; IRE_12 = Irish samples from 2012; SCO = Scotland; S_ENG = South England; MED = Mediterranean; SA = South Africa; PAC = New Zealand; NWA = Northwest Atlantic. Maps were created in ArcGIS v.10 (http://www.esri.com/arcgis/about-arcgis).

Global DAPC cluster analysis showed that throughout all *a priori* groups, individuals had a higher probability of assignment to a population other than where they were originally sampled when using the function *find.clusters*. Consequently, a DAPC of the putative populations showed that none of the global samples clustered tightly based on their sampling origin, although samples from IRE_12, NWA and the MED were most different from the NEA or PAC region (Fig. 6).

Similarly, a PCoA of Nei’s genetic distances by population separated IRE_12, SA, NWA and MED on the principal axis (53.31%) from the remaining NEA and PAC samples (Supplementary Fig. S9). Throughout all analyses, it became apparent that individuals sampled in the Pacific were genetically close to the centroid of all samples. Finally, Bayesian inference using Structure could not detect any clustering in the data set (*K* = 1), maintained using *locpriors* and without *a priori* information on population origin.

### Genetic diversity

Table 2 shows that sample sizes on a global scale were unbalanced and mean number of alleles per site corresponded with the number of individuals sampled, except for IoM, which showed a lower number of alleles than IRE (for NEA-specific diversity measures see Supplementary Table S9). Allelic richness, a measure of genetic diversity adjusted for sample size, ranged from 4.042 (IRE_12) to 4.295 (MED). The MED and PAC samples showed heterozygote deficit (high *F*_IS_), with IRE and PAC samples significantly out of HWE; probably a consequence of combining temporal samples of opportunistically collected individuals.

### Northeast Atlantic effective population size *Ne*

When computing *N*_*e*_, rare allele frequencies were excluded to avoid bias (P_crit_ < 0.02), an approach not adversely affecting precision^31^. Most *N*_*e*_ estimates exceeded the sample size used in the estimates (Supplementary Table S8). Applying the *N*_*e*_ estimator to all (n = 97) individuals from the IoM temporal data set gave an estimated *N*_*e*_ (using P_crit_ = 0.02) of 382 individuals (parametric CIs = 241–830). If this point estimate were to be corrected by 0.74–0.86 to account for age-structure, the upwardly adjusted *N*_*e*_ comes to 516, which would suggest over 800 (*N*_*e*_:N ratio used = 0.6) individuals frequenting IoM waters.

For a separate analysis, IoM samples were combined with SCO samples, resulting in no accurate estimates of *N*_*e*_ (*N*_*e*_ = infinite) and parametric CIs from 3239 – infinity (P_crit_ = 0.02). Using the same adjustments as above, a minimum of *N*_*e*_ = 3239 would suggest at least 4,000 individuals frequenting IoM and SCO waters.

## Discussion

### Genetic evidence supports inter-annual site-specific re-encounters

Our analyses of the largest NEA temporal (2009–2014) set of basking shark DNA samples collected to date, provides the first supporting genetic evidence for site-specific re-encounters in the basking shark, with 18% of individuals re-sampled inter/multi-annually at or near the same location. These findings accord with previous evidence from photo-ID re-sightings within a region^32^, mark-recapture methods at a localised site^29^, and more recent satellite tracking^18^.

Notably, we resampled seven individuals in close proximity (<20 km) to their first sampling location, suggesting inter-annual returns to these areas. While estimates of basking shark energetic requirements suggest over-wintering at the northerly limits of our survey is possible, inshore habitats of seasonally enhanced productivity are exploited only temporally, after which they move to other favourable, usually deeper habitats^14,33,34^. Recent satellite tracking identified summer (July-October) residency within the Sea of the Hebrides^18^, followed by various migration strategies into more southerly latitudes, showing a displacement range of 455km-2354km based on archival tags^17^. Neither study suggests individuals are year-around inshore residents found within a few kilometres of their tagging location. Rather, corroboration of all available evidence makes a compelling case that basking sharks leave an area to return on an inter- or perhaps multi-annual basis. Inter-annual returns of two satellite-tagged sharks to the Scottish MPA^18^ are concordant with wider genetic re-sampling of individuals from both Scottish and Manx (Isle of Man) waters within a few kilometres of where they were first encountered, often after several years. Our complementary evidence from Scottish, Irish and Manx aggregation sites suggests individuals may exhibit inter-annual site fidelity in the NEA, although data remains insufficient to suggest site fidelity on a population-level. To determine the scale and frequency of inter-annual basking shark returns at a population level, the calculated probability of recaptures would need to exceed that of random expectations^20^, where the chance of resampling an individual is inversely proportional to population size.

The scale, frequency and nature of return movements in elasmobranchs is complex and has been variously attributed to gestation^12^, pupping^35^, mating^36^ or feeding^37^. Seasonality in prey availability is a common driver of migratory movement, and repeated use of an area for feeding is considered ‘feeding site fidelity’^38,39^. A strong association with thermal fronts and chlorophyll *a* in NEA shelf seas and low inter-annual variability of seasonally persistent fronts likely encourages basking sharks to return on an inter-annual basis^19^. Already documented in large-bodied lamniform sharks returning seasonally to areas supporting abundant prey^1,6^, this phenomenon is also observed in other large planktivores, such as the whale shark^37^ and the giant oceanic manta, *Mobula birostris*^40^.

Confirmation of inter-annual site fidelity can inform seasonal/dynamic protection measures around aggregation sites, particularly in wide-ranging marine predators. Yet, compared with some marine mammals^41^, there is little knowledge of the spatial and temporal variation of such events in planktivorous elasmobranchs, and reacquisition of individuals between years at the same site requires continuous and unambiguous quantification.

### Population structure in the NEA

Seasonal affinity to oceanic regions has potential to promote population structure, but not if there are sufficient migrants to counter genetic drift. It is unclear if documented ocean-scale movement^15,16^ of basking sharks is for gestation purposes or a consequence of mating dispersal (involving temporarily/permanently leaving a home range). However, only the latter could lead to effective dispersal and hence gene flow, as it requires dispersers to reproduce in a ‘new’ location^42^. Determining the number of migrants required to homogenize population structure, counteracting drift and selection, is a contentious issue. The basking shark, like the similarly long-lived whale shark^25^, would require only a few breeding migrants every 2–4 years to homogenize population structure.

Our temporal sampling regime in the NEA afforded an opportunity to examine the regional population structure of this species in unprecedented detail. Subtle but significant temporal structuring of populations in the NEA was apparent from estimates of *G*_ST_, with early-season sharks sampled off SW Ireland in 2012 (IRE_12) differentiated from most other temporal samples. Further, IRE12 shows a significantly higher mean group relatedness than expected, and departure from HWE consistent with the notion of genetically discrete kin groups moving *en masse*.

These observations are reminiscent of sympatric subpopulations sharing the same spatially, but not temporally, defined locations^43^. Whereas the migratory route of IRE_12 individuals cannot be ‘retraced’, their genetic similarity to earlier samples of NWA sharks in all analyses of population differentiation (Supplementary Figs. S8, S9) supports the possibility of trans-Atlantic migration. Such movements of marine fauna^44,45^ may be facilitated by the North Atlantic gyre system, following the north-easterly flow of the Gulf Stream and the North Atlantic Current. Basking sharks may follow a similar route to forage along the highly productive Sub Polar Front. The lowest conformation to HWE in Scottish waters recorded in 2012 may reflect aggregations of genetically more discrete migrant groups.

With certain caveats, our analysis of recent genetic migration rates and directionality in the NEA may suggest an asymmetrical pattern of immigration into the Irish Sea (IoM sampling area). However, as some assumptions of the inference model were not met (low, constant migration rates and high F_ST_ differentiation^46^), these findings should be interpreted as a merged Irish Sea (IoM) sample set representing all NEA individuals at one time point, rather than source-sink population dynamics. That the Irish Sea (IoM), increasingly influenced by anthropogenic activity, could represent an important transit corridor during seasonal movements within the NEA deserves further investigation. Concordant with this interpretation is the limited but significant differentiation between merged temporal samples from Irish (excluding IRE_12) and Scottish waters (Figs. 2 & 6).

### Relatedness within NEA aggregations

The distribution of genotypes in time and space, inferred from analysis of adult group relatedness, suggests basking sharks surfacing together in the NEA are on average more related than expected by chance. Within-group relatedness was highest in early-season (April) samples, as represented by IRE_12 and IRE_14. All-female groups had higher kinship than all-male groups or mixed assemblages (Supplementary Fig. S7); however sample sizes preclude inference about patterns of relatedness and gender.

Following Jacoby *et al*.^47^, ‘aggregation’ refers to individuals drawn to the same resource for food or other habitat requirements in the absence of evidence suggesting social interactions. Spatio-temporal co-occurrence of individuals, and potential social interactions, may arise through shared behavioural strategies including spatial segregation during certain life stages^12^, collective movements^48^, and aggregation around a resource^11,13^ or habitat^18^. Site-attached behaviours, such as aggregations due to patchy resource distribution, characteristic of basking sharks, periodically bring individuals together.

Understanding the spatial and temporal patterns of aggregations can contribute to long-term conservation efforts^12,49^. While inferring relatedness from genotypic data alone remains challenging and should be used with caution^50,51^, in species with little opportunity for direct observation^52^ it provides some insight into the potential mechanisms underlying fine-scale behavioural processes with long-term ecological consequences^53^. Estimates of relatedness and inbreeding have by definition a large randomly-mating and non-inbred reference population and should be understood as measures relative to this reference^54^. Theoretically, when sampling free-ranging animals from a larger population, individuals are assumed to be drawn at random and are therefore unrelated; making the mean estimate of relatedness close to zero, suggesting samples contain few or no relatives^51^. Kin groupings could be a consequence of inter-annual site fidelity to seasonal foraging opportunities^19^. Ultimately, a tendency to aggregate seasonally increases the probability of kin encounters, possibly facilitating social interactions or transmission of information between conspecifics and kin. When individual learning (‘asocial’, i.e. trial and error) is costly, social learning from conspecifics or kin can be advantageous, particularly in heterogeneous environments^55^. Larger, more experienced basking sharks may be more proficient at locating patchy food than juvenile sharks, due to ontogenetic differences in habitat selection^34^, as recently demonstrated in other marine predators^56^. Therefore, social information transfer and the potential to learn optimal foraging may benefit kin in loose aggregations^57^. However, that not all aggregations comprised significant kin groups, and overall exhibit a lack of evidence for inbreeding, raises the possibility of assortative mating in the basking shark. Differentiating between kin and unrelated conspecifics might be a mechanism to tolerate or seek kin during foraging, but to avoid breeding with close relatives. Kin recognition has been shown in salmonids^58^, and an experimental study demonstrated male guppies’ reproductive investment (courtship) was reduced when placed with siblings^59^. Although it is unknown if basking sharks are similarly capable, olfaction-mediated pairing behaviour has been suggested as a mechanism in sharks^60^.

### Global connectivity

Long-distance migration is common in mobile, long-lived species occurring at many spatial and temporal scales^7^. Our analysis of available samples from outside the NEA suggests lack of population differentiation, often interpreted as evidence of global panmixia, which contrasts with subtle but significant structuring within the NEA. Ocean-scale tracking studies indicate that neither the Mid-Atlantic Ridge nor tropical waters are barriers to migration^15,16^. Basking sharks appear to have a broad thermal range^14^ and are therefore relatively unrestricted by temperature. Considering their average cruising speed^3^, individuals could travel a straight-line distance of over 30,000 km annually. This supports the possibility of ocean-wide dispersal, suggesting movement among all sample locations is not beyond their known physical/physiological capabilities.

Similar movement analyses of the more thermally restricted whale shark inferred they existed as a global ‘meta-population’^4^. However, population genetic analyses using microsatellite and mitochondrial DNA found significant structure between Indo-Pacific and Atlantic populations^25^. This study emphasised inadequate population samples and marker choice had influenced previous interpretations of whale shark population structure^61^. Similarly combining our Pacific samples across years could have degraded statistical differentiation of the Pacific and NEA populations; although the possibility that their apparent genetic similarity has its historic origins in trans-Arctic migration during an ice-free period^62^ cannot be refuted. Adequate sampling, by contrast, revealed weak population structure in both species; the genetically most discrete whale sharks from the isolated Holbox Island, Gulf of Mexico^61^, and similar weak differentiation of the temporally discrete IRE_12 sharks.

Consequently, at a larger scale the prevailing view that basking sharks exhibit panmixia, lacking global population structure, cannot be rejected immediately by our microsatellite analysis of combined temporal samples collected worldwide. However, that these samples, where large enough for analysis to be meaningful, depart significantly from HWE suggests they could comprise individuals from genetically discrete temporal populations, biasing downward estimates of differentiation. This inference is supported by our findings of some temporal genetic differentiation and unexpectedly high levels of relatedness within basking shark aggregations in the NEA. Therefore, as in earlier studies of whale sharks^61^, it remains questionable whether lack of differentiation between small, unrepresentative and perhaps most importantly, arbitrary ‘population’ samples, collected opportunistically from strandings and fishery captures over tens of years and across five oceanic regions, really can indicate high contemporary genetic connectivity and lack of population structure.

Effective population size. Effective population size (*N*_*e*_) influences long-term evolutionary processes and estimates of *N*_*e*_ are generally lower than census size reflecting reproductive variability^27^. Most of our estimates for the NEA were at least three-fold the sample size. Larger sample sizes meant *N*_*e*_ could not be estimated or had infinite confidence limits, suggesting that the linkage disequilibrium (LD) method could not distinguish larger *N*_*e*_ from infinity, possibly reflecting a lack of true, independent populations.

Aerial surveys based on the Bay of Fundy, Canada, estimated the total population size of eastern Canada as 6512 (CI: 4040–11886)^63^. In accordance with Hoelzel *et al*.^21^ (*N*_*e*_ of 8200 animals worldwide, calculated from mitochondrial markers), and based on our microsatellite-derived *N*_*e*_ estimates, the number of basking sharks in NEA waters are unlikely to exceed 10,000 individuals.

Despite efforts to reduce bias in our estimates, two requisites of the LD model, closed populations and random mating, were violated. Therefore, our sampling design and adjustments likely reflect a per-generation, local *N*_*e*_, rather than number of breeders within the population. Finally, *N*_*e*_/N_c_ ratios in large-bodied elasmobranchs are relatively unexplored, but a suggested ratio similar to those of marine and terrestrial mammals seems appropriate, considering elasmobranchs share similarly slow life history traits^64^. Merged Irish Sea samples from four successive years produced an estimated *N*_*e*_ of 382 (95% CI = 241–830), inferring >800 individuals frequent the Irish Sea (*N*_*e*_:N ratio used after correcting point estimate = 0.6), similar to a local abundance estimate from neighbouring Scottish waters of 985 (95% CI = 494–1683)^29^.

### Implications

We report site-specific re-encounters of individuals, which supports suggestions of inter-annual site fidelity^17,18^, and revealed high levels of gene flow among NEA aggregations. However, we have also found an early-season sample (IRE_12) whose genetic composition was temporally heterogeneous. There was a tendency for a spatial association of kin groups, perhaps drawn together by prey availability following ocean scale migration. Findings which do not contradict the inference of some level of global connectivity. Maintaining the integrity of these aggregations of related individuals undertaking focused seasonal return movements to feeding sites together, supports the case for area protection; while highlighting the global risks accruing to the basking shark’s characteristic adherence to sites and transit routes crossing multiple jurisdictions. Our analysis of migration rates associated with the Irish Sea, an area of intense marine renewable development, exemplifies the problem of protection during transit between aggregation sites. Nevertheless, knowledge of the spatial ecology and population connectivity of highly migratory species can inform spatial planning. Identification of aggregation ‘hotspots’, areas associated with the presence of persistent fronts, informed the recent proposal for a basking shark MPA in Scottish waters^10^. Its potential importance is underscored by observations of suspected mating activity within summer aggregations^10,11^.

Finally, our study suggests an important corollary to the well documented catch rate declines of the 1950s to 1990s associated with area-focused basking shark fisheries^3,23^. Evidence of relatedness, most marked in early season samples, suggests kin association may be characteristic. Activities depleting kin groups can erode selectable genetic variation rapidly; an important consideration in a slowly reproducing long lived species exhibiting limited genetic diversity.

## Material and Methods

### Sample collection, DNA extraction and amplification

A total of 460 basking shark samples (skin/mucus) were collected from five oceanic regions during the last two decades (1994–2014). Tissue samples from dead, mostly stranded or trawl bycaught specimens were obtained opportunistically from the Northwest Atlantic (n = 12), South Africa (n = 5), the Mediterranean (n = 11) and New Zealand, representing the South Pacific (n = 38). The majority (>85%) of samples were collected from the Northeast Atlantic (NEA; n = 394) using mainly mucus swabs of free-swimming basking sharks^65^. All mucus sampling methodology was approved by, and conducted with the knowledge of the Wildlife and Conservation Division of the Isle of Man Government, Scottish Natural Heritage licence (13921 and 58660) and the UK Home Office ASPA licence (PPL 30/2975; University of Exeter). Three common aggregation sites within the NEA region were sampled repeatedly between 2009 and 2014 to investigate inter- and intra-annual connectivity within and among the waters of the Isle of Man (n = 118), Ireland (n = 124), and the West Coast of Scotland (n = 124). In addition, 22 samples were collected from individuals in the Moray Firth, Scotland, in 2013, representing the only North Sea sampling location. Finally, six individuals were sampled from Southern England off Cornwall, 1994–2004. Associated data, such as sampling location, total length, sex and dorsal fin-ID were acquired when possible and samples for DNA extraction were preserved in Analytical Reagent Grade absolute ethanol. Total genomic DNA was extracted using proteinase K digestion and standard phenol-chloroform procedures. DNA concentration was quantified using a fluorometer (Qubit, Invitrogen) and its quality assessed by electrophoresis on a 1% agarose gel stained with ethidium bromide.

All samples were genotyped for 22 dinucleotide microsatellites, including 19 loci from Lieber *et al*.^30^ (*Cmax01*–19) and three loci from Noble *et al*.^66^ (*1HA5, 1HC2, 1HF4;* two of the original five loci showed severe stuttering), following the multiplex design described in Lieber *et al*.^30^. Products were amplified in a 2-µl PCR reaction, including 1 µl (30–80 ng) DNA (air dried), 1 µl primer mix (forward and reverse primer at 0.2 or 0.4 mM) and 1 µl QIAGEN Multiplex PCR Master Mix (including HotStar *Taq* DNA polymerase) with the following profile: 95 °C for 15 min, followed by 35 cycles of 94 °C for 30 s, 56 °C for 90 s, 72 °C for 60 s and finally 60 °C for 6 min. PCR products were diluted (1:160) and separated on an ABI 3730 48-well capillary DNA Analyser using ROX GS500 size standard (Applied Biosystems Inc.). Genotypes were scored using the GENEMAPPER v3.7 software.

### Microsatellite characterisation

To assess genotyping error rates, a random set of 87 individuals (19% of the entire data set) was re-amplified and re-scored at all 22 loci, producing 1,914 multilocus genotypes (22 loci × 87 individuals). The mean error rate per locus was calculated as the ratio between the number of single-locus genotypes including at least one allelic mismatch and the number of replicated single-locus genotypes. Each microsatellite locus was checked for scoring errors, allelic dropout and null alleles using Microchecker 2.2.3^67^. Individuals that failed to amplify at more than six loci were removed from the data set.

Observed and expected heterozygosities and mean number of alleles per locus were calculated in GenAlEx 6.5^68^. The probability of randomly drawing two individuals from the population sharing identical multilocus genotypes was calculated in GenAlEx using both, P_ID_(unbiased) and P_ID_(sibs), to determine the upper and lower boundaries of probability of identity^69^. Fisher’s exact tests of Hardy-Weinberg equilibrium (HWE) using a Markov Chain Monte Carlo (MCMC) approach with 10,000 iterations and tests for linkage disequilibrium (LD) per locus and putative population were performed in GenePop 4.2^70^. Significant values for multiple comparisons were adjusted by calculating false discovery rate (FDR) adjusted *p*-values.

As population genetic assessments require neutral loci, an *F*_ST_ outlier method using the software Lositan^71^ was applied to evaluate if all loci were neutral. The method evaluates the relationship between *F*_ST_ and expected heterozygosity (H_E_) to identify outliers that have disproportionately high or low *F*_ST_ values compared to neutral expectations. The first run estimates the mean neutral *F*_ST_ using all loci, a consecutive run uses only putative neutral loci to compute neutral *F*_ST_, and following the simulation, Lositan then reports the estimated selection status (balancing selection, neutral, positive selection) of each locus. Lositan was run with 100,000 simulations using the infinite allele model, using the ‘neutral mean *F*_ST_’ and ‘Force mean *F*_ST_’ options. In order to assess if the inclusion or exclusion of suspected non-neutral loci had an effect on estimates of population differentiation, genetic pairwise distances were generated with and without loci under selection, and with only ‘neutral’ loci as identified by Lositan.

### Matching analysis and site fidelity

Due to the non-destructive sampling procedures used on live animals, there was a possibility that the same individuals were sampled repeatedly. Matching analyses were performed in GenAlEx to assess intra- and inter annual genetically confirmed re-encounters of individuals within sites (sites = IoM, IRE, SCO, MF) or between years. Exact matches and near-matches (up to three mismatching loci) were considered as genetically confirmed re-encounters, genetic recaptures, and removed for subsequent population analyses. Near-matches with >3 mismatching loci were assessed one by one to evaluate if mismatches were likely a result of allelic drop-out or stutter. When available, genetically confirmed re-encounters were also evaluated with dorsal fin-ID photographs, size and sex of the individual.

### Relatedness analysis within northeast Atlantic aggregations

Mean group relatedness based on the Queller and Goodnight (1989)^72^ relatedness coefficient was determined by 1,000 bootstraps and 95% confidence determined by 999 permutations to test the null hypothesis of ‘no differences across populations’ using GenAlEx. To allow for a detailed investigation into basking shark genetic group structure with respect to relatedness, a genetic marker-based approach was applied. For this, a subset of spatio-temporal groupings (n = 55, 2–14 individuals per group, average group size = 4) from IoM, SCO (the West Coast and the Moray Firth), and IRE was used to further test the null hypothesis that groups represent, on average, random associations with respect to relatedness. Defined as spatio-temporal sampling events, groups were defined as affiliations between two or more individuals that were within the same cluster at the surface when sampling commenced. In short, simulations were performed using the allele frequencies of the data set to generate 200 simulated pairs for each degree of relatedness (full-sibs, half-sibs, parent-offspring, unrelated) using four relatedness estimators (dyadic likelihood method: di^73^); and three moment estimators: LR^74^; QG^72^; and W^75^ as implemented in the R package ‘related’ v.1.0 using the function *compareestimators*^76^. Following evaluation of the four estimators, the degree of resolution that should be expected (using the chosen moment-based estimator LR, denoted ‘LynchRD’ hereafter and the likelihood estimator di, denoted ‘DyadML’) was analysed using another simulation implemented in the *familysim* function in ‘related’. Relatedness estimates per group were calculated as the average within-group relatedness (R) derived from all pairwise comparisons within the group using the current sample set as the underlying reference population^51^. Following the simulations, variances of LynchRD and DyadML for pairs of relatedness indicated that they performed well in differentiating unrelated pairs from related ones. To compare estimators, R was calculated using both, the dyadic likelihood method DyadML, which constraints estimates to the range of [0, 1], and the moment estimator LynchRD whose range also yields negative estimates^74^, using the *grouprel* function in ‘related’ with a 1000 iterations to compare the average observed R value with the expected distribution. Similar to estimates derived from the software ‘Storm’ v. 2.0^77^, the *grouprel* function calculates the average pairwise relatedness values within each observed (pre-defined) group, resulting in an average within-group relatedness estimate. The expected distribution of average within-group relatedness values is then generated by shuffling individuals between groups while keeping each group size constant using a 1000 Monte Carlo simulations. This generates 1000 iterations of each group, where each iteration is one realisation of the expected relatedness value of the data set. The observed mean relatedness is then compared to the distribution of average simulated values to test the null hypothesis of groupings being random associations with respect to relatedness. Both relatedness estimators used above assume non-inbred individuals. However, possible non-random mating due to demonstrated patterns of site fidelity could violate the assumption of a large outbred population. Therefore, average inbreeding coefficients were computed per group (by averaging the inbreeding coefficient per individual within a group), using the *coancestry* function in ‘related’. Additionally, within-group R computations were repeated with the option ‘allow inbreeding’ using DyadML (denoted as ‘DyadML_F’) to account for possible inbreeding using 1000 iterations.

### Effective population size (N_e_)

Contemporary *N*_*e*_ (estimates apply to the time period encompassed by the sample) was estimated using the bias-corrected single-sample molecular method based on linkage disequilibrium (LD)^31^ as implemented in NeEstimator v2^78^. Estimates were made excluding alleles with frequencies less than 0.02 and 0.05 (denoted as ‘P_crit_’). The analysis was performed on eight global sites (excluding S_ENG due to small sample size) and then repeated on a set of merged NEA samples. For the latter, Irish sharks were excluded to avoid any downward bias in *N*_*e*_ caused by a potential Wahlund effect (R. Waples, personal communication), as using samples from a ‘meta’-population with local subdivision can violate the main assumptions of the LD method. Merged SCO and IoM temporal samples resulted in 207 individuals of all age-classes. Simulations have shown that estimates of *N*_*e*_ performed best for mixed-age samples (if the number of age classes approximated generation length), thereby approaching *N*_*e*_ per generation^79^. Remaining downward-bias due to age structure was corrected according to mixed-age samples of species whose reproductive capacities might be most comparable to basking sharks (grizzly bear = 0.74; dolphin = 0.79; elephant seal = 0.86^80^). Therefore, an adjustment of 1/(0.74–0.86) = 1.16–1.35 was used to increase the LD point estimate. Finally, the *N*_*e*_/N ratio (effective population size to adult census population size ratio) which needs to be interpreted with caution, was based on estimates for mammals: *N*_*e*_/N = 0.6–1.0 (*N*_*e*_/N ratio in fish: 0.4–1.0)^80^. A rather conservative *N*_*e*_*/*N ratio of 0.6 was considered to be applicable to basking sharks in the absence of reliable estimates of life history parameters and was used to infer adult census population size based on NEA samples.

### Measures of genetic differentiation

Unbiased estimators of differentiation *D*_ST_^81^ and *G*_ST_^82^ were calculated for 15 NEA putative populations based on sampling site and year to test for site fidelity, and to assess whether temporal samples from the same site could be merged for subsequent global comparisons. *G*_ST_ (a generalized form of *F*_ST_ for multi-allelic loci) is considered the most appropriate metric when mutation rates are low relative to migration rates, as might reasonably be expected in basking sharks, and patterns of population structure are driven primarily by migration^81^. Bias-corrected 95% confidence intervals using 1,000 bootstraps in the diveRsity (function ‘fastDivPart’) package^83^ in R^84^ were estimated to test concordance of differentiation statistics. Using FSTAT v2.9.3.2^85^, *F*_ST_ pairwise comparisons were also calculated for the 15 NEA putative populations, for comparative purposes only and reflecting this metric’s widespread historic use; however, when using highly variable microsatellites, levels of heterozygosity within populations can be underestimated. Significant p-values for multiple comparisons were adjusted using a sequential Bonferroni correction. After merging populations within the NEA into five populations (IoM, SCO, IRE, IRE_12, S_ENG), based on either sampling site or G_st_ differentiation level (in the case of IRE_12). *G*_ST_, *D*_ST_ and *F*_ST_ pairwise comparisons were repeated, including the five NEA sites in addition to four global sites (MED, SA, NWA, S_PAC), resulting in nine global, putative populations (for sample sizes, see Supporting Information, Table S2).

A Discriminant Analysis of Principal Components (DAPC) in the Adegenet v1.3.4. package^86^ in R was implemented using all nine putative populations. When genetic clusters could not be defined using the clustering algorithm *k-means* and the *find.clusters* function, a DAPC was implemented using the nine putative populations as prior groups. The data were first transformed using principal component analysis, followed by a Discriminant Analysis on 60 retained principal components (cross-validation was performed as an optimisation procedure to identify the number of principal components with the lowest root mean squared error) using all eight discriminant functions. DAPC is a dimension-reduction approach and is constructed using alleles which most reflect the between-group variance, thereby reducing variance within putative populations. In the resulting DAPC graph, individuals were represented as dots and the groups as inertia ellipses.

A DAPC was repeated using merged samples from the NEA (SCO, all years; IoM, all years; IRE, 2009, 2010, 2011, 2014; IRE_12, 2012) to investigate if individuals sampled from Irish waters were genetically more distinct compared to SCO and IoM individuals. A principal coordinate analysis (PCoA) visualising Nei’s pairwise genetic distances^87^ was performed in GenAlEx for the NEA samples as well as for all nine putative, global populations.

Another Bayesian cluster algorithm was used as implemented in Structure v2.3^88^ to infer possible undetected *posteriori* genetic clusters using the nine global putative populations. Run length parameters included 500,000 MCMC iterations (after a 100,000 iteration burn-in period) with assumed genetic clusters (*K*) set from 1–10 (15 runs per *K*) using the admixture ancestry model with correlated allele frequencies. The analysis was repeated with the same parameters, however this time, *a priori* sampling locations as prior information using the LOCPRIOR parameter setting^89^ were considered to detect possible weak population structure signals in this highly mobile shark. In order to determine the most likely *K* (Δ*K*), the Evanno method^90^ was applied in Structure Harvester^91^.

### Contemporary migration rate and direction in the NEA

To estimate the direction and rate of contemporary migration (**m)** over the last several generations, a Bayesian approach based on Markov Chain Monte Carlo (MCMC) methods to estimate the posterior probabilities of the migration matrix among sub-populations was used as implemented in BayesAss v.3.0^46^. The sub-populations were based on the NEA aggregation sites, consisting of merged samples from the IoM (n = 97), SCO (n = 133), IRE (n = 89) and the temporal sample of IRE_12 (n = 18) in order to assess migration rates and directionality within the NEA. BayesAss uses multi-locus genotypes and does not require the populations to be in migration drift or Hardy–Weinberg equilibrium. As suggested by the software authors, more accurate results are obtained when the number of proposed changes for the parameters m (migration rate), P (allele frequencies), and F (inbreeding coefficient) yield an acceptance rate between 20% and 60%. Initial runs used the following parameter settings: m = 0.3, P = 0.15 and F = 0.2. However, parameters were adjusted in the final run to achieve acceptance rates <60% and to ensure optimal mixing, using the following parameters: m = 0.5, P = 0.6 and F = 0.6, resulting in acceptance rates ranging from 0.38–0.57%. The number of iterations for the MCMC analysis was set to 5 × 10^−06^ with a burn-in period of 5 × 10^−05^ (number of MCMC iterations discarded before sampling commences) and a thinning interval of 1,000 iterations. Convergence was diagnosed through multiple runs and by plotting the log-probability of the iterations (see Supplementary Fig. S9).

### Genetic diversity

Genetic diversity measures were calculated per global sampling site and within the NEA. Allelic richness, using the rarefaction method (with a minimum sample size of seven diploid individuals) was calculated in FSTAT. Wright’s inbreeding coefficient *F*_IS_ (a measure of the reduction in heterozygosity within a subpopulation), number of different alleles, mean number of alleles, private and effective alleles, observed and expected heterozygosity, and mean group relatedness (r) were computed in GenAlEx. Exact tests for HWE per site using a Markov Chain Monte Carlo (MCMC) approach with 10,000 iterations were performed in GenePop.

## Supplementary information

Supplementary information.
