# Supplementary material for: Spatio-temporal genetic tagging of a cosmopolitan planktivorous shark provides insight to gene flow, temporal variation and site-specific re-encounters
Source: Sci Rep. 2020 Feb 3;10:1661. doi: 10.1038/s41598-020-58086-4 (PMC6997447; doi:10.1038/s41598-020-58086-4)
Supplement: Supplementary file 1 — Supplementary information. [file 41598_2020_58086_MOESM1_ESM.docx]

# Supplementary information

**Spatio-temporal genetic tagging of a cosmopolitan planktivorous shark provides insight to gene flow, temporal variation and site-specific re-encounters**

Lilian Lieber, Graham Hall, Jackie Hall, Simon Berrow, Emmett Johnston, Chrysoula Gubili, Jane Sarginson, Malcom Francis, Clinton Duffy, Sabine P. Wintner, Phil D. Doherty, Brendan J. Godley, Lucy A. Hawkes, Matthew J. Witt, Suzanne M. Henderson, Eleonora de Sabata, Mahmood S. Shivji, Deborah A. Dawson, David W. Sims, Catherine S. Jones, Leslie R. Noble

**Supplementary Table S1:** Initial basking shark sample sizes per site and final sample sizes shown following matching analyses (pairs of samples were regarded as matches when genotypes mismatched at three or fewer loci).

| Site | Initial sample sizes | N taken out after matching analysis | Sample sizes* after matching analysis |
| --- | --- | --- | --- |
| IoM09 | **9** | 1 | 8 |
| IoM10 | **35** | 8 | 27 |
| IoM11 | **12** | 1 | 11 |
| IoM12 | **38** | 9 | 29 |
| IoM13 | **24** | 2 | 22 |
| IRE09 | **15** | 4 | 11 |
| IRE10 | **34** | 2 | 32 |
| IRE11 | **23** | 5 | 18 |
| IRE12 | **21** | 3 | 18 |
| IRE14 | **31** | 3 | 28 |
| MF13 | **22** | 2 | 20 |
| SCO12 | **62** | 9 | 53 |
| SCO13 | **57** | 2 | 55 |
| SCO14 | **5** | 0 | 5 |
| S_ENG | **6** | 0 | 6 |
| MED | **11** | 0 | 11 |
| SA | **5** | 1 | 4 |
| PAC | **38** | 0 | 38 |
| NWA | **12** | 1 | 11 |
| TOTAL | 460 | 53 | 407 |

Sampling locations include: IoM= Isle of Man, IRE= Ireland, MF= Moray Firth, Scotland, SCO= West Scotland, with year indicated accordingly and S_ENG= South England, MED= Mediterranean; SA= South Africa; PAC= New Zealand, NWA= Northwest Atlantic N, number of individuals, *, Samples sizes used for measures of genetic differentiation, probability of identity and microsatellite characterisation.

**Supplementary Table S2:** Final sample sizes of the nine global, putative populations following matching and relatedness analyses and the total removal of 53 individuals. These samples were taken for all measures of differentiation and genetic diversity estimates.

| Sample sizes for 9 putative populations | Site |
| --- | --- |
| 97 | IoM |
| 89 | IRE |
| 18 | IRE_12 |
| 133 | SCO |
| 6 | S_ENG |
| 11 | MED |
| 4 | SA |
| 38 | PAC |
| 11 | NWA |

Global sampling locations include: IoM= Isle of Man, IRE= Ireland, IRE_12= Ireland (from 2012 only) SCO= West Scotland and the Moray Firth, S_ENG= South England, MED= Mediterranean; SA= South Africa; PAC= New Zealand, NWA= Northwest Atlantic.

**Supplementary Table S3:** Characterisation of 19 microsatellite loci in 407 basking sharks

| Locus | N | k | error rate | H_O_ | H_E_ | PIC | HWE | F(Null) |
| --- | --- | --- | --- | --- | --- | --- | --- | --- |
| *1HA5* | 399 | 11 | 0.0115 | 0.591 | 0.632 | 0.602 | NS | 0.036 |
| *1HC2* | 353 | 6 | 0.0115 | 0.470 | 0.483 | 0.403 | NS | 0.007 |
| *1HF4* | 404 | 6 | 0.0115 | 0.636 | 0.632 | 0.571 | NS | -0.010 |
| *Cmax01* | 405 | 3 | 0.0115 | 0.506 | 0.502 | 0.377 | NS | -0.005 |
| *Cmax02* | 406 | 3 | 0.0115 | 0.224 | 0.240 | 0.212 | NS | 0.034 |
| *Cmax03* | 405 | 5 | 0 | 0.652 | 0.650 | 0.578 | NS | 0.001 |
| *Cmax04* | 407 | 7 | 0.0115 | 0.742 | 0.711 | 0.662 | NS | -0.026 |
| *Cmax05* | 407 | 5 | 0.0115 | 0.538 | 0.517 | 0.400 | NS | -0.022 |
| *Cmax06* | 391 | 15 | 0.0115 | 0.754 | 0.779 | 0.749 | NS | 0.017 |
| *Cmax07* | 394 | 18 | 0 | 0.802 | 0.879 | 0.867 | NS | 0.044 |
| *Cmax08* | 365 | 9 | 0 | 0.795 | 0.767 | 0.736 | NS | -0.021 |
| *Cmax09* | 407 | 4 | 0.0115 | 0.570 | 0.555 | 0.492 | NS | -0.018 |
| *Cmax10* | 406 | 5 | 0.0115 | 0.761 | 0.706 | 0.654 | NS | -0.040 |
| *Cmax11* | 407 | 7 | 0.0115 | 0.796 | 0.745 | 0.700 | NS | -0.035 |
| *Cmax15* | 401 | 13 | 0.023 | 0.738 | 0.790 | 0.762 | NS | 0.033 |
| *Cmax16* | 405 | 6 | 0.0115 | 0.669 | 0.692 | 0.641 | NS | 0.017 |
| *Cmax17* | 407 | 10 | 0.0115 | 0.828 | 0.815 | 0.792 | NS | -0.008 |
| *Cmax18* | 406 | 15 | 0 | 0.865 | 0.873 | 0.859 | NS | 0.006 |
| *Cmax19* | 405 | 6 | 0.0115 | 0.560 | 0.594 | 0.544 | NS | 0.024 |
| Mean | 399 | 8 | 0.0097 | 0.658 | 0.661 | 0.611 | NS | 0.002 |

Characteristics per locus calculated in CERVUS v. 3.0.3*, where N, number of individuals genotyped; k, number of alleles; error rate, based on re-genotyping of 87 individuals where mean error rate per locus was calculated as the ratio between the number of single-locus genotypes including at least one allelic mismatch and the number of replicated single-locus genotypes ; H_O_, observed heterozygosity; H_E_, expected heterozygosity; PIC, Polymorphic Information Content, HWE, significance testing for Hardy-Weinberg equilibrium (HWE) where NS=not significantly different from HWE; F(Null), estimated frequency of null alleles. *Kalinowski, ST, Taper, ML & Marshall, TC (2007) Revising how the computer program CERVUS accommodates genotyping error increases success in paternity assignment. *Molecular Ecology* **16**: 1099-1106.

**
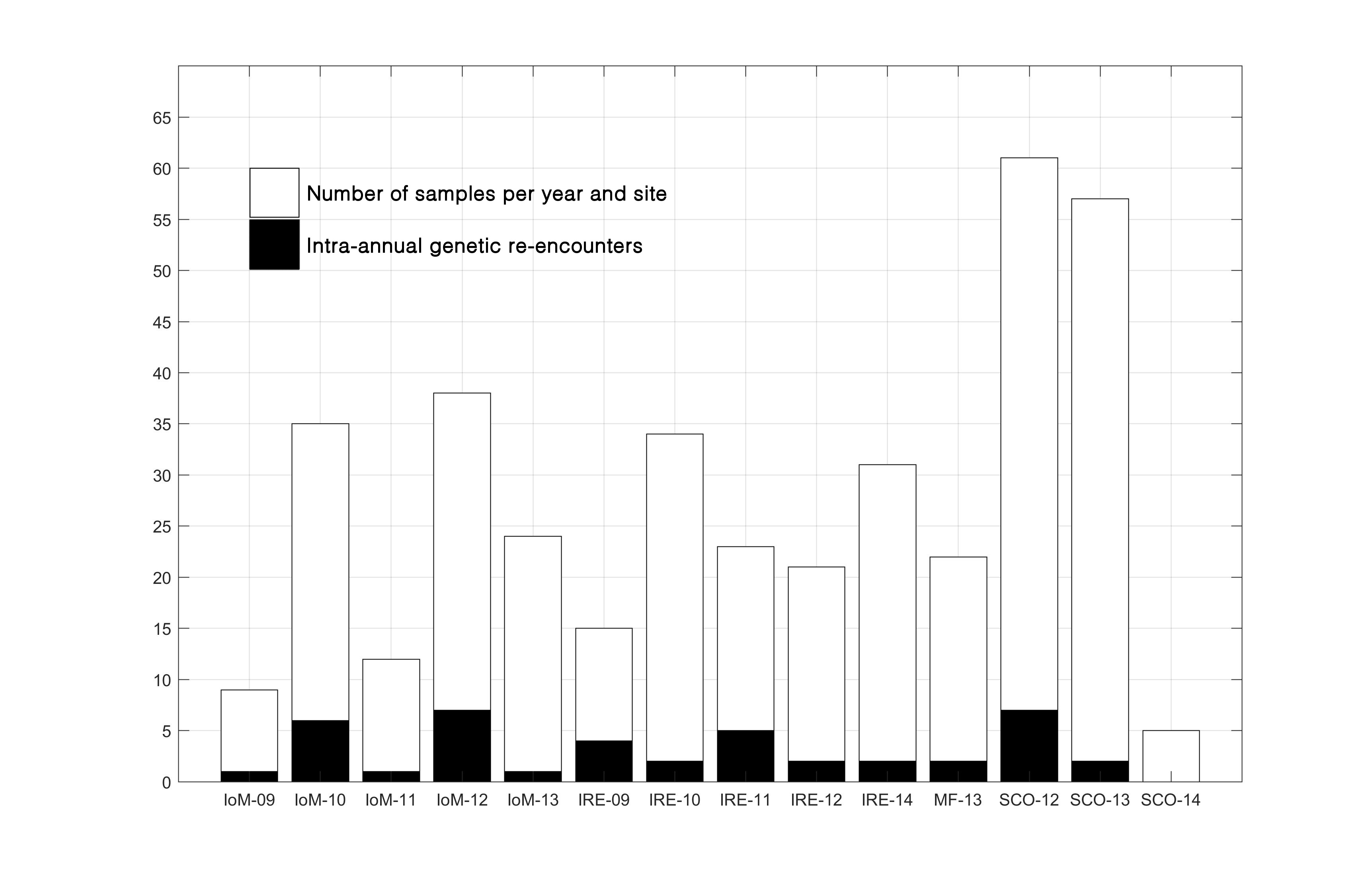
**

**Supplementary Figure S1**: Number of individuals sampled and intra-annual genetically confirmed re-encounters (individuals sampled repeatedly within a site and year) on the y-axis, and sites (where IoM =Isle of Man, IRE= Ireland, MF= Moray Firth, SCO= Scotland; and numbers represent sampling years post 2000) on the x-axis.

**
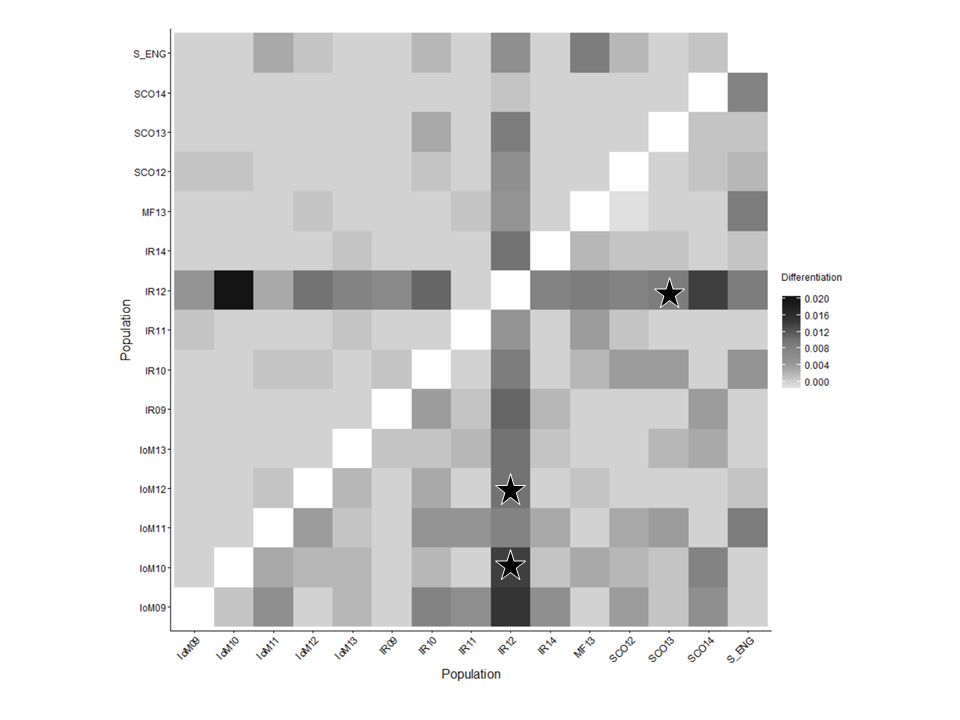
**

**Supplementary Figure S2**: Pairwise *D*ST (above diagonal) and *G*ST (below diagonal) genetic differentiation among Northeast Atlantic (NEA) samples (IoM =Isle of Man; SCO= Scotland; MF= Moray Firth; IR= Ireland; S_ENG= South England; where the numbers represent sampling years post 2000). Stars (🟋) indicate significant comparisons at 95% confidence intervals using 1,000 bootstraps.

**Supplementary Table S4**: Wright’s pairwise *F*ST comparisons between 15 Northeast Atlantic temporal samples below the diagonal, with annotated significance level at the 5 % level (bold) above. None were significant using the Bonferroni-corrected level (α = 0.000476). IoM =Isle of Man; SCO= Scotland; MF= Moray Firth; IR= Ireland; S_ENG= South England; where the numbers represent sampling years post 2000.


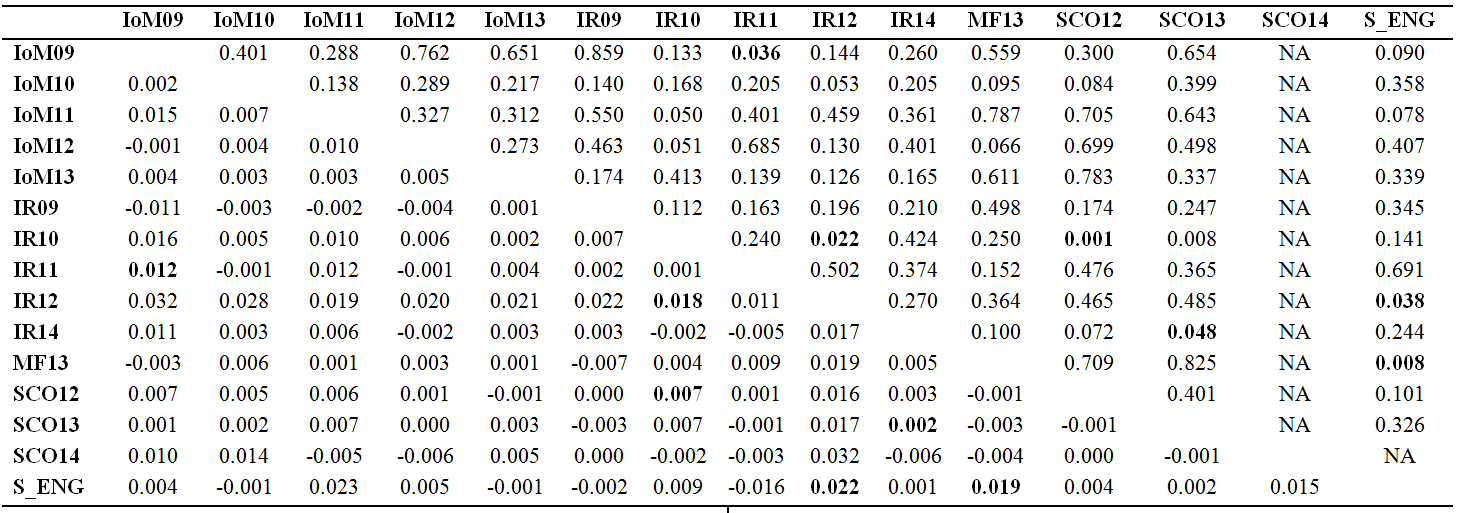


**Supplementary Figure S3**: Principal Coordinates Analysis (PCoA) of Nei’s pairwise genetic distances in the Northeast Atlantic sample (IoM = Isle of Man; SCO= Scotland; MF= Moray Firth; IR= Ireland; where the numbers represent sampling years post 2000). SCO_14 and S_ENG have been excluded due to very low sample sizes.

**Supplementary Table S5**: Means of the posterior distributions of **m** (along with their standard deviations), the migration rate into each population, are shown. The source ‘populations’ from which individuals migrated are listed in the columns, while the ‘populations’ from which individuals were sampled are listed in the rows. Values along the diagonal (italic & bold) are the proportions of individuals derived from the source populations each generation, while migration rates >0.10 are shown in bold. F, given in the columns, denotes the population-specific inbreeding coefficient as estimated in BayesAss. Sampling locations include: IoM= Isle of Man, IRE= Ireland, IRE_12= Ireland (from 2012 only), SCO= West Scotland.

|  | IoM (F=0.0174 ± 0.0080) | IRE (F=0.2652 ± 0.2087) | IRE_12 (F=0.3589 ± 0.2512) | SCO (F=0.1614 ± 0.1153) |
| --- | --- | --- | --- | --- |
| IoM | ***0.9529*** (± 0.0254) | **0.3115** (± 0.0118) | **0.2632** (± 0.0369) | **0.3205** (± 0.0073) |
| IRE | 0.0135 (± 0.0111) | ***0.6726*** (± 0.0061) | 0.0210 (± 0.0205) | 0.0043 (± 0.0043) |
| IRE_12 | 0.0033 (± 0.0034) | 0.0037 (± 0.0037) | ***0.6844*** (± 0.0171) | 0.0024 (± 0.0024) |
| SCO | 0.0303 (± 0.0235) | 0.0122 (± 0.0101) | 0.0314 (± 0.0270) | ***0.6729*** (± 0.0054) |


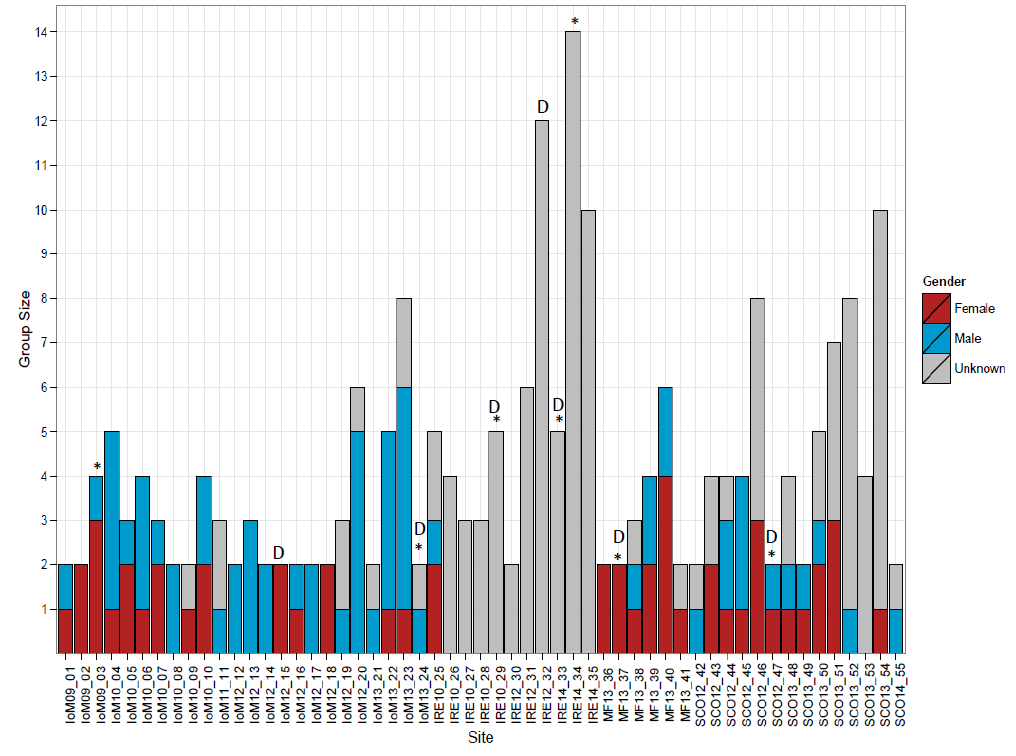


**Supplementary Figure S5**: Number of individuals and gender composition (where available) of basking shark groups (n=55) sampled around the Isle of Man (IoM, 2009-2013), Ireland (IRE, 2010-2012, 2014), the Moray Firth, Scotland (MF, 2013) and the West Coast of Scotland (SCO, 2012-2014). Groups that are significantly (p<0.05) more related than expected by chance based on a 1000 MCMC iterations using the relatedness estimators LynchRD and Dydad_F (accounting for inbreeding) are marked with an asterisk ‘*’, or with a ‘D’, respectively.

**
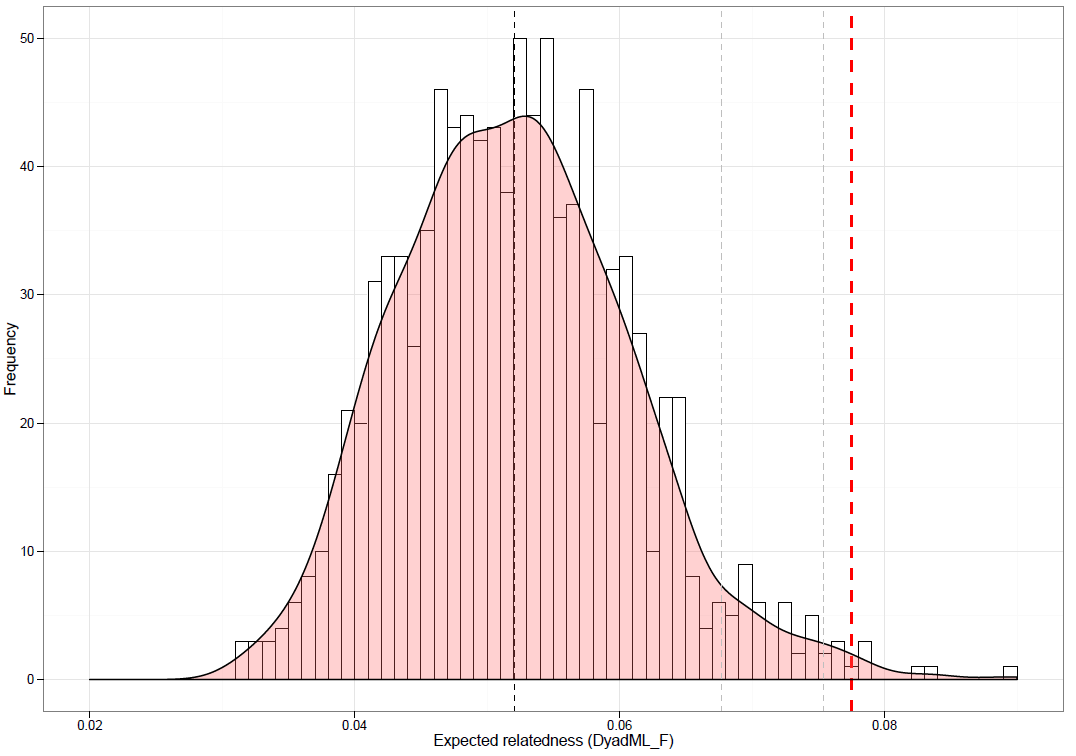
**

**Supplementary Figure S6**: Histogram with kernel density curve of observed, average within-group relatedness based on the likelihood method ‘DyadML’^74^ with inbreeding taken into account. Average within group relatedness examined across all 55 groups (red dashed line= 0.0775, p<0.008) and expected pattern of relatedness using a 1000 iterations. The iteration’s mean (=0.0521) is shown as a dark grey dashed line and the 95% (0.0677) and 99% (0.0754) quantiles shown as a light grey dashed line, respectively.


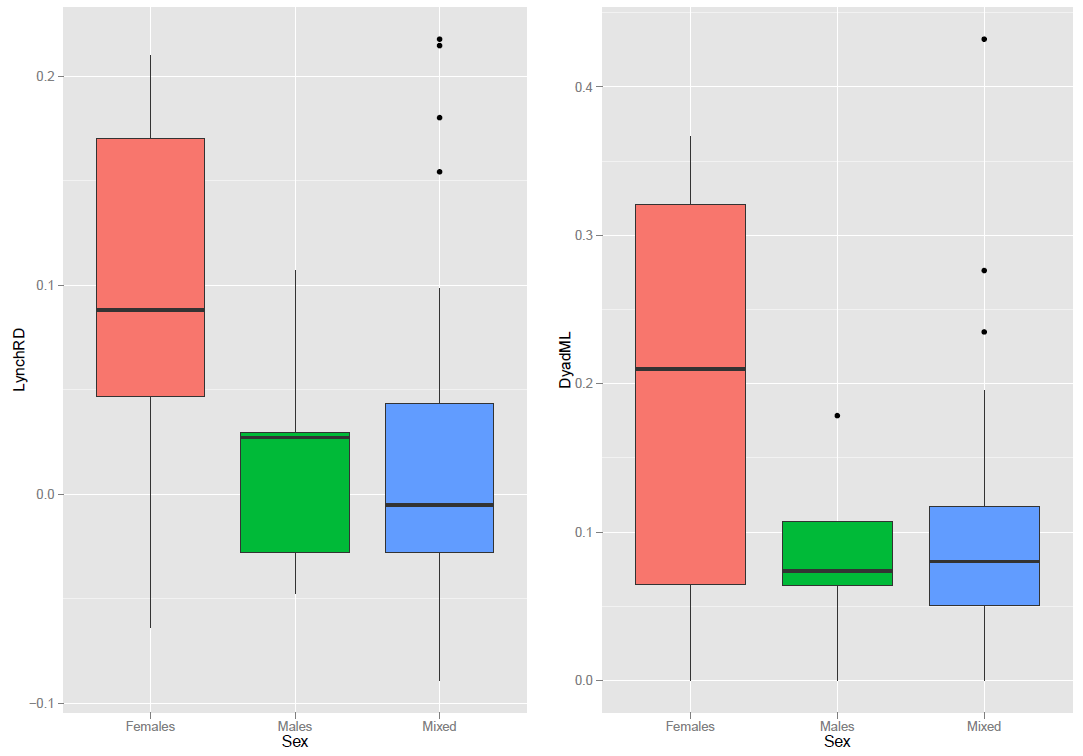


**Supplementary Figure S7**: Boxplots showing the variance in within-group relatedness of basking shark groups (n=55) by sex based on LynchRD (left) and DyadML (right). There were only five all-female groups (‘Females’), five all-male groups (‘Males’) and 45 mixed-sex groups (‘Mixed’; also applied to groups of unknown sexes). Sampling sites include the Isle of Man (IoM, 2009-2013), Ireland (IRE, 2010-2012, 2014), the Moray Firth, Scotland (MF, 2013) and the West Coast of Scotland (SCO, 2012-2014).


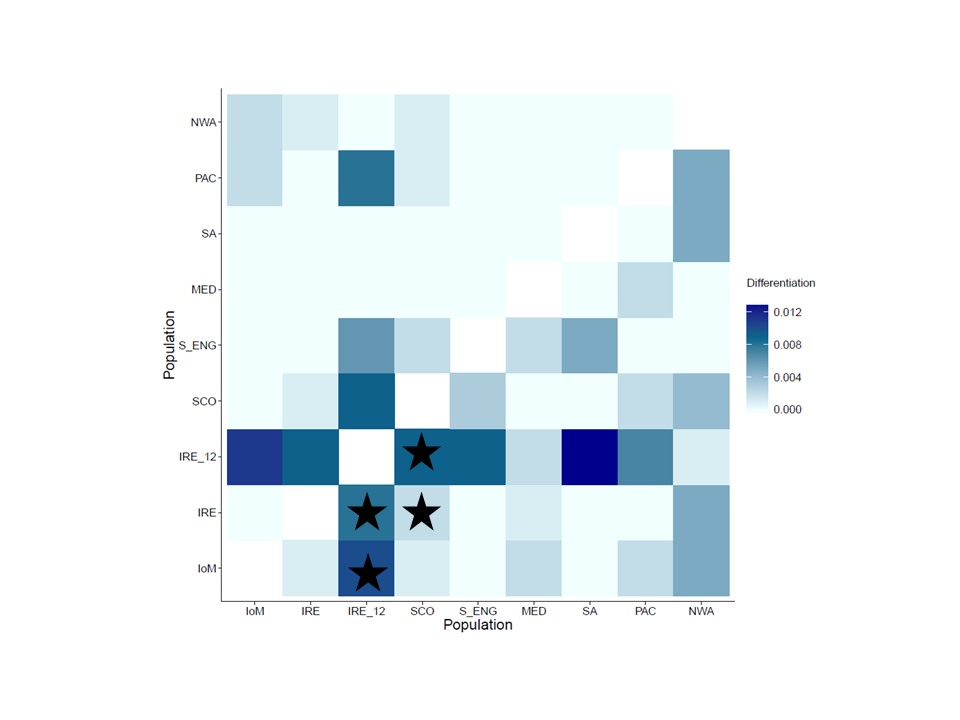
**Supplementary Figure S8­**: Pairwise *D*_ST_ (above diagonal) and *G*_ST_ (below diagonal) genetic differentiation among global samples with annotated (🟋) significant comparisons (when 95% confidence limits were not bounded by zero after a 1,000 bootstraps). IoM=Isle of Man (n=97); IRE= Ireland (n=89); IRE_12= Irish samples from 2012 (18); SCO= Scotland (n=133); S_ENG= South England (n=6); MED= Mediterranean (n=11); SA= South Africa (n=4); PAC= New Zealand (n=38); NWA= Northwest Atlantic (n=11).

**Supplementary Table S7:** Wright’s pairwise *F*ST comparisons between nine global samples below the diagonal, with annotated significance level at the 5 % level (bold) above. None were significant using the Bonferroni-corrected level (α = 0.001389). Where IoM= Isle of Man; IRE= Ireland; IRE_12= Irish samples from 2012; SCO= Scotland; S_ENG= South England; MED= Mediterranean; SA= South Africa; PAC= New Zealand; NWA= Northwest Atlantic.

|  | IoM | IRE | IRE_12 | SCO | S_ENG | MED | SA | PAC | NWA |
| --- | --- | --- | --- | --- | --- | --- | --- | --- | --- |
| IoM |  | **0.046** | 0.122 | 0.296 | 0.167 | 0.093 | 0.946 | 0.043 | 0.313 |
| IRE | **0.002** |  | 0.235 | **0.003** | 0.299 | 0.685 | 0.956 | 0.129 | 0.429 |
| IRE_12 | 0.020 | 0.016 |  | 0.461 | **0.035** | 0.324 | 0.690 | 0.283 | 0.586 |
| SCO | 0.001 | **0.003** | 0.017 |  | 0.107 | 0.307 | 0.982 | 0.072 | 0.429 |
| S_ENG | 0.001 | -0.001 | **0.022** | 0.005 |  | 0.076 | 0.289 | 0.474 | 0.139 |
| MED | 0.005 | 0.001 | 0.004 | 0.000 | 0.001 |  | 0.975 | 0.536 | 0.388 |
| SA | -0.007 | -0.007 | 0.033 | -0.011 | 0.021 | -0.028 |  | 0.878 | 0.260 |
| PAC | 0.005 | 0.001 | 0.013 | 0.004 | -0.008 | 0.002 | -0.004 |  | 0.414 |
| NWA | 0.010 | 0.009 | 0.005 | 0.008 | 0.007 | -0.004 | 0.022 | 0.009 |  |


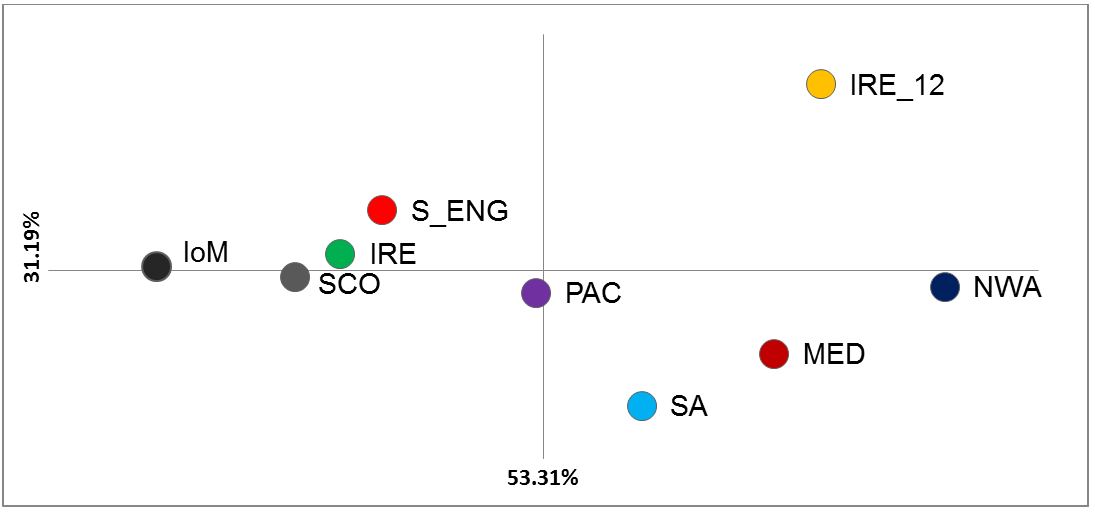


**Supplementary Figure S9**: Principal Coordinates (PCoA) analysis on Nei’s pairwise genetic distance matrix between global samples. Axis 1 explains 53.31% and axis 2, 31.19% of the variation in the data; where IoM= Isle of Man (n=97); IRE= Ireland (n=89); IRE_12= Irish samples from 2012 (18); SCO= Scotland (n=133); S_ENG= South England (n=6); MED= Mediterranean (n=11); SA= South Africa (n=4); PAC= New Zealand (n=38); NWA= Northwest Atlantic (n=11).

|  | Lowest Allele  Frequency | Estimated  N_e_ | 95% CIs  (Parametric) | 95% CIs  (Jack–knife) |
| --- | --- | --- | --- | --- |
| IoM | 0.05 | 529.1 | 272.8 – 3861.7 | 253.3 – 43784.8 |
|  | 0.02 | 381.6 | 241.4 – 830.2 | 237.6 – 871.7 |
| IRE | 0.05 | 386 | 212 – 1549.8 | 188.6 – 6016.3 |
|  | 0.02 | 623.1 | 297.1 – Infinite | 274.2 – Infinite |
| IRE_12 | 0.05 | 342.3 | 55.4 – Infinite | 53.8 – Infinite |
|  | 0.02 | 111.3 | 44.4 – Infinite | 44.6 – Infinite |
| SCO | 0.05 | Infinite | 2678.2 – Infinite | 1398.9 – Infinite |
|  | 0.02 | Infinite | 2344.7 – Infinite | 1505.7 – Infinite |
| S_ENG | 0.05  0.02 | Infinite  Infinite | 81.8 – Infinite  81.8 – Infinite | Infinite – Infinite  Infinite – Infinite |
| MED | 0.05 | Infinite | 41.6 – Infinite | 48.7 – Infinite |
|  | 0.02 | Infinite | 35.1 – Infinite | 41.5 – Infinite |
| SA | 0.05 | 2.9 | 1.5 – 25.4 | 1.9 – 9.4 |
|  | 0.02 | 2.9 | 1.5 – 25.4 | 1.9 – 9.4 |
| PAC | 0.05 | 208 | 91.1 – Infinite | 79.1 – Infinite |
|  | 0.02 | 522.9 | 154.4 – Infinite | 132 – Infinite |
| NWA | 0.05 | 8 | 4.4 – 14.4 | 4.8 – 13.1 |
|  | 0.02 | 8.7 | 4.9 – 15.9 | 5.5 – 14.2 |

**Supplementary Table S8**: N_e_ estimator outputs per sampling site with P_crit_=0.05 and 0.02 denoted as lowest allele frequency used.

Sampling locations include: IoM= Isle of Man, IRE= Ireland, IRE_12= Ireland (from 2012 only), SCO= West Scotland, S_ENG= South England, MED= Mediterranean; SA= South Africa; PAC= New Zealand, NWA= Northwest Atlantic.

**Supplementary Table S9:** Genetic diversity statistics derived from 19 microsatellite loci across all unique Northeast Atlantic, temporally collected basking shark samples.

| Sampling site & year sampled | N | N_dA_ | N_A_ | P_A_ | A_R_ | N_E_ | H_O_ | H_E_ | HWE | F | r |
| --- | --- | --- | --- | --- | --- | --- | --- | --- | --- | --- | --- |
| IoM_2009 | 8 | 80 | 4 | 0.000 | NA | 3.022 | 0.658 | 0.604 | 0.803 | -0.067 | 0.020 |
| IoM_2010 | 27 | 108 | 6 | 0.000 | 4.091 | 3.775 | 0.702 | 0.677 | 0.446 | -0.039 | -0.044 |
| IoM_2011 | 11 | 90 | 5 | 0.105 | 3.967 | 3.177 | 0.684 | 0.617 | 0.878 | -0.113 | 0.039 |
| IoM_2012 | 29 | 109 | 6 | 0.000 | 3.879 | 3.344 | 0.675 | 0.637 | 0.411 | -0.069 | 0.022 |
| IoM_2013 | 22 | 106 | 6 | 0.000 | 3.965 | 3.341 | 0.651 | 0.639 | 0.132 | -0.015 | 0.012 |
| IRE_2009 | 11 | 95 | 5 | 0.105 | 4.201 | 3.405 | 0.637 | 0.638 | 0.699 | -0.007 | -0.037 |
| IRE_2010 | 32 | 111 | 6 | 0.053 | 4.047 | 3.551 | 0.661 | 0.663 | 0.181 | 0.010 | -0.024 |
| IRE_2011 | 18 | 98 | 5 | 0.053 | 3.974 | 3.424 | 0.678 | 0.649 | 0.716 | -0.051 | -0.017 |
| IRE_2012 | 18 | 98 | 5 | 0.105 | 3.842 | 3.117 | 0.658 | 0.609 | 0.115 | -0.064 | **0.101** |
| IRE_2014 | 28 | 106 | 6 | 0.158 | 3.873 | 3.339 | 0.656 | 0.646 | 0.541 | -0.018 | -0.002 |
| MF_2013 | 20 | 105 | 6 | 0.000 | 4.072 | 3.504 | 0.671 | 0.644 | 0.959 | -0.040 | -0.003 |
| SCO_2012 | 53 | 122 | 6 | 0.105 | 3.977 | 3.529 | 0.642 | 0.648 | 0.218 | 0.003 | 0.007 |
| SCO_2013 | 55 | 122 | 6 | 0.000 | 3.990 | 3.597 | 0.645 | 0.648 | 0.452 | 0.452 | 0.007 |
| SCO_2014 | 6 | 70 | 4 | 0.000 | NA | 2.778 | 0.618 | 0.572 | 0.975 | -0.053 | 0.073 |
| S_ENG | 5 | 67 | 4 | 0.000 | NA | 2.704 | 0.695 | 0.580 | 0.998 | -0.203 | 0.047 |

Sampling locations include: IoM= Isle of Man, IRE= Ireland, MF= Moray Firth, Scotland, SCO= West Scotland, S_ENG= South England, with year indicated accordingly; N, sample size; N_dA_, number of different alleles; N_A_, mean number of alleles across 19 loci; PA, number of private alleles; A_R_, allelic richness based on 6 diploid individuals; N_E_, number of effective alleles; H_O_, observed heterozygosity; H_E_, expected heterozygosity; HWE, p-value for Hardy-Weinberg equilibrium probability test; F, Fixation index (average inbreeding coefficient of subpopulations relative to the total population), r= mean group relatedness determined by 999 bootstraps where * indicates if different across putative populations determined by 999 permutations. NA= Non-applicable (samples excluded from allelic richness estimate due to low sample size).


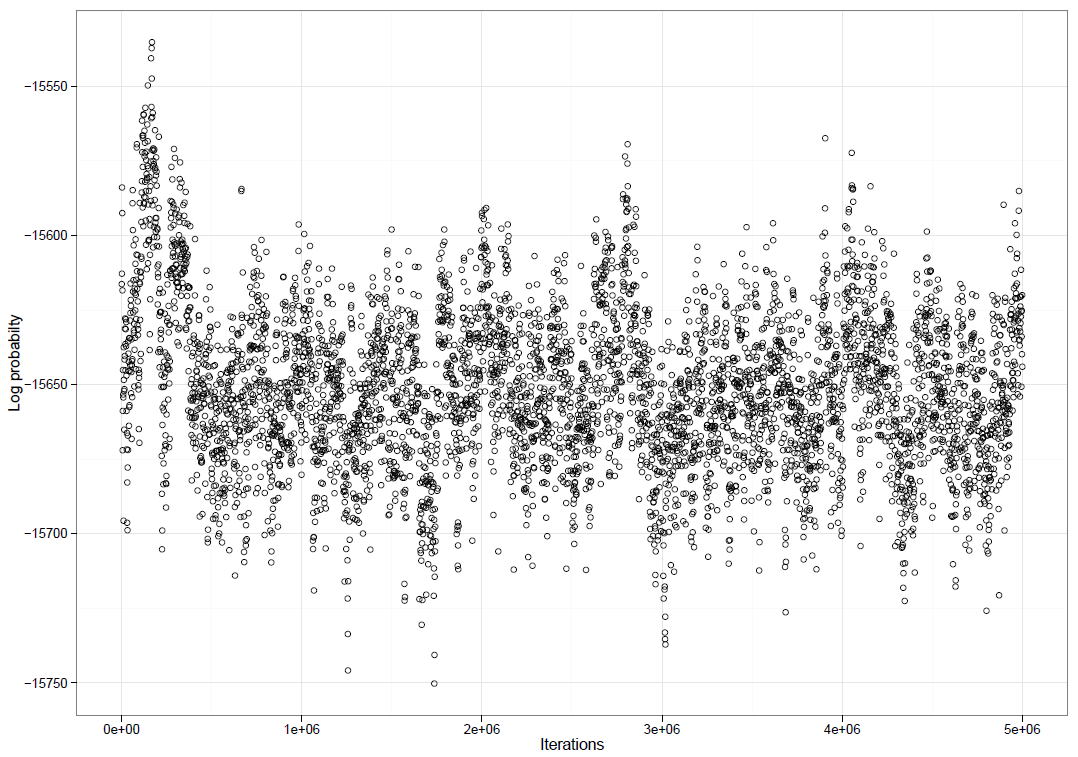


**Supplementary Figure S9**: Scatterplot of the trace file for log-probability analysis in BayesAss after 5,000,000 sample iterations, 50,000 burn-in iterations and a thinning interval of 1000 iterations, used for convergence diagnosis. The log-probability initially increases during the burn-in, but then oscillates regularly around a plateau, indicating that the MCMC chain has converged, sampling the whole parameter space.
